# Modulation of the immunogenic landscape in colorectal cancer by mitochondrial methylation-controlled J protein

**DOI:** 10.1186/s43556-026-00466-9

**Published:** 2026-05-11

**Authors:** Maram Ahmed, Shoja M. Haneefa, Aftab Alam, Suresh K. Kali, Ashraf Al-Sbiei, Ghada Bashir, Alia Al-Bawardi, Ienas Idriss, Yassir A. Mohamed, Batoul Khlaifat, Heba T. Naser, Lena Labania, Rifat Hamoudi, Uday Kishore, Samir Attoub, Mercedes Rincon, Khalil B. Ramadi, Maria J. Fernandez-Cabezudo, Basel K. Al-Ramadi

**Affiliations:** 1 Department of Medical Microbiology and Immunology, College of Medicine and Health Sciences, United Arab Emirates University, Al Ain, United Arab Emirates; 2 Department of Biochemistry and Molecular Biology, College of Medicine and Health Sciences, United Arab Emirates University, Al Ain, United Arab Emirates; 3 Department of Pathology, College of Medicine and Health Sciences, United Arab Emirates University, Al Ain, United Arab Emirates; 4 Division of Engineering, New York University Abu Dhabi, Abu Dhabi, United Arab Emirates; 5 Research Institute for Medical and Health Sciences, University of Sharjah, P.O. Box 27272, Sharjah, United Arab Emirates; 6 Department of Clinical Sciences, College of Medicine, University of Sharjah, P.O. Box 27272, Sharjah, United Arab Emirates; 7 Division of Surgery and Interventional Science, University College London, London, United Kingdom; 8 Department of Veterinary Medicine, College of Agriculture and Veterinary Medicine, United Arab Emirates University, Al Ain, UAE; 9 Zayed Center for Health Sciences, United Arab Emirates University, Al Ain, United Arab Emirates; 10 Department of Pharmacology & Therapeutics, College of Medicine and Health Sciences, United Arab Emirates University, Al Ain, United Arab Emirates; 11 Immunology & Microbiology Department, University of Colorado Anschutz Medical Campus, Aurora, USA; 12 Tandon School of Engineering, New York University, New York, NY 11201 USA; 13 Grossman School of Medicine and NYU Langone Health, New York University, New York, NY 11201 USA; 14 ASPIRE Precision Medicine Research Institute Abu Dhabi, United Arab Emirates University, Al Ain, United Arab Emirates; 15 Present address: Department of Internal Medicine, VCU School of Medicine, Virginia Commonwealth University, Richmond, VA 23298 USA

**Keywords:** Tumor microenvironment, Metabolic reprogramming, Cancer immunogenicity, Tumor-infiltrating lymphocytes

## Abstract

The tumor microenvironment (TME) plays a pivotal role in cancer progression by orchestrating interactions between cancer cells and surrounding immune and non-immune cells through metabolic reprogramming. Methylation-controlled J protein (MCJ; also known as DNAJC15) is a negative regulator of mitochondrial respiration, but its role in tumor metabolism remains unclear. To study the role of tumor-intrinsic MCJ expression, we generated MCJ-deficient cancer cell lines using siRNA and performed a comprehensive analysis of their characteristics in comparison with MCJ-expressing parental cells. MCJ deficiency resulted in enhanced mitochondrial respiration and ATP production. Despite normal growth in vitro, MCJ-deficient tumor cells exhibited severly curtailed growth in immunocompetent mice. However, these cells grew comparably to controls in T-cell-deficient athymic mice, indicating immune-mediated suppression. Tumors lacking MCJ exhibited increased immune cell infiltration and immunogenicity, as determined by immunohistochemistry and flow cytometry. Transcriptomic analysis revealed a metabolic shift from glycolysis-dominant and hypoxic conditions to a state favoring oxidative phosphorylation, which correlated with upregulation of immune-related pathways and enhanced anti-tumor immune responses. Bioinformatic analysis conducted to study the correlation between the level of MCJ expression and tumor functions in human colorectal cancer revealed that low MCJ expression correlated with increased tumor mutational burden, microsatellite instability, and immune cell infiltration, demonstrating the clinical relevance of MCJ expression in the context of colon adenocarcinoma. These findings suggest MCJ as a promising target for metabolic immunotherapy in cancer.

**Supplementary Information:**

The online version contains supplementary material available at 10.1186/s43556-026-00466-9.

## Introduction

The tumor microenvironment (TME) is a dynamic and intricately regulated milieu that comprises not only cancer cells but also a diverse group of non-malignant cells, extracellular matrix components, and blood vessels [1]. Tumor progression is regulated by the metabolic activity of tumor cells as well as other cells in the TME, including endothelial cells, fibroblasts and immune cells, a process collectively known as metabolic reprogramming [2]. The interplay between the various cellular components within the TME contributes to a complex metabolic landscape that fosters tumor progression and metastasis. Central to this operation is the mitochondria which produce the majority of the chemical energy required to fuel the metabolic demands within normal as well as tumor cells. The movement of electrons by the various complexes of the electron transport chain (ETC) is critical for ATP production. This continuous flow of electrons through the ETC provides the metabolic plasticity that is required for tumor progression [3]. In this context, understanding the molecular players that modulate metabolic reprogramming in the TME and the pathways through which tumor metastasis is influenced are crucial for developing targeted therapeutic strategies.

Methylation-controlled J (MCJ) protein, a member of the DnaJ heat shock protein family, has garnered attention for its role in cancer chemoresistance [4–6]. MCJ is an endogenous negative regulator of complex I of the ETC and is regulated epigenetically by methylation at 3 CpG sites located in the proximal promoter region of the gene [4]. MCJ is located on the inner mitochondrial membrane and acts in mitochondrial biogenesis [7, 8]. Abundant expression of MCJ is observed in liver, heart and kidney tissues as well as in immune cells, particularly CD8 T cells and, to a lesser extent, macrophages [8–11]. Loss of MCJ has been shown to confer resistance to chemotherapeutic drugs in ovarian and breast cancer cells [5, 6], most likely through the increased functional activity of drug efflux transporters [12]. Moreover, low MCJ expression on tumor cells has been shown to predict poor response to chemotherapy among breast cancer patients [4]. While the precise functions of MCJ in cancer biology are still being elucidated, its central involvement in regulating the activity of the ETC suggests that it may play a key role in metabolic reprogramming within the TME.

In the current study, we examined the role of MCJ in tumor growth by comparing the in vitro and in vivo growth characteristics of MCJ-positive and MCJ-deficient tumor cells, using the MC38 colorectal adenocarcinoma model. Whole genome transcriptomic analysis was carried out using RNASeq to delineate the differentially expressed genes (DEGs) and functional pathways affected by MCJ deficiency. MC38 cancer cells lacking MCJ expression exhibited severely impaired tumor growth in a syngeneic mouse model. We demonstrate that the impaired tumor growth of MCJ-deficient cells is due to their enhanced immunogenicity. Collectively, our data indicate that MCJ-dependent alterations in metabolic reprogramming within the TME can regulate the immunogenicity of cancer cells. These findings set the stage for a detailed investigation of the role of MCJ in shaping the metabolic landscapes within the TME, offering insights into novel avenues for therapeutic intervention in cancer.

## Results

### Derivation and characterization of MCJ-deficient MC38 cells

**Fig. 1:**
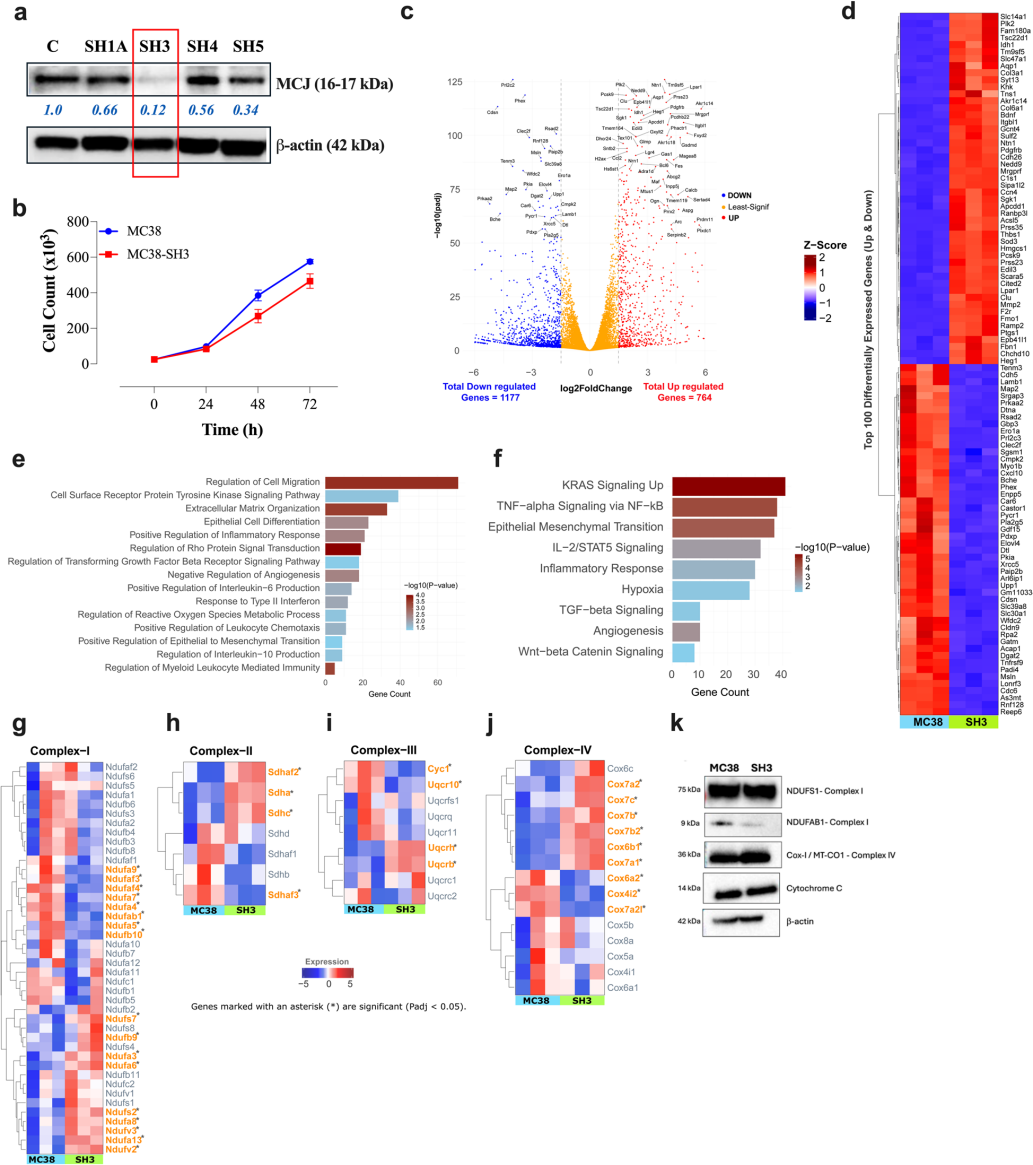
Derivation and characterization of MCJ-deficient MC38 transfectants. **a** Western blot analysis of MCJ expression in normal MC38 cells (control lane) and 4 stable cell lines following transfection with the MCJ siRNA construct. **b** Proliferative capacity of MC38-SH3 cells compared to parental MC38 cells as determined by trypan blue manual cell counting. **c** A volcano plot illustrating differentially expressed genes (DEGs) in MC38-SH3, showcasing log2-fold change against statistical significance (-Log10(P-adj)). Blue dots (left side) signify down-regulated gene expression, red dots indicate up-regulation, and orange dots represent genes with the least significant expression differences. **d** A heatmap showing the top 50 differentially expressed genes (both upregulated and downregulated) across all samples. The horizontal axis represents the sample groups (MC38 and SH3), while the vertical axis displays the expression levels of the top genes. Red indicates upregulated genes, and blue indicates downregulated genes. **e** Over-representation analysis of DEGs to identify key biological processes (GO). Bar length represents the total gene count, and color intensity (from dark to light) reflects the -log10(*p*-value). **f** MSigDB over-representation analysis for pathway enrichment. Bar length indicates the total gene count, and color gradient (dark to light) corresponds to the -log10(*p*-value). **g**-**j** Heatmaps showing differential expression of genes associated with Complex I, Complex II, Complex III, and Complex IV subunits in MC38 and MC38-SH3 cells. Genes marked with an asterisk (*) and highlighted in orange are statistically significant (Padj < 0.05). **k** Western blots of representative components of ETC complexes. Data shown in panels **a**, **b **and **k** are representative of 2–3 experiments each

To investigate the role of MCJ in regulating tumor growth, stable MCJ-deficient transfectants of the MC38 colorectal adenocarcinoma cell line were generated using siRNA-mediated knockdown. MC38 is a well-characterized, relatively immunogenic tumor model that shares key features with human colorectal cancer [13]. Multiple stable transfectants exhibiting varying levels of MCJ expression were obtained (Fig. 1a). Among these, clone MC38-SH3, which exhibited ~88% reduction in MCJ expression compared to parental cells, was selected for subsequent studies (Fig. 1a).

MCJ deficiency did not affect in vitro proliferation, as both MC38 and MC38-SH3 cells exhibited comparable proliferation rates and viability (Fig. 1b). To further investigate the impact of MCJ deficiency, global transcriptomic profiling was performed (Fig. 1c). This analysis identified 1,941 DEGs, including 764 upregulated and 1177 downregulated (Log2FC > 1.5) in MCJ-deficient cells (Fig. 1c). A heatmap showing the top 100 DEGs is presented in Fig. 1d, with the complete dataset provided in Table S1.

Pathway enrichment analysis using Gene Ontology Biological Processes (GO:BP) and the MSigDB Hallmark (MH) gene sets revealed significant alterations in pathways related to inflammatory cytokine signaling, leukocyte chemotaxis, epithelial-mesenchymal transition (EMT), angiogenesis, hypoxia, and cell migration (Fig. 1e-f; Tables S2-S3). These findings suggest that MCJ deficiency induces transcriptional reprogramming consistent with a more inflamed “hot” tumor phenotype [14].

Further analysis of ETC-associated genes revealed differential expression across complexes I-IV (Fig. 1g-j; Table S4). Among complex I, the largest of the ETC complexes with 42 subunits, there were 9 upregulated and 8 downregulated genes in MC38-SH3 cells (Fig. 1g). Similar patterns were observed in Complexes II, III and IV subunits (Fig. 1h-j). Validation of these results was carried out on selected proteins, namely NDUFS1 and NDUFAB1, by Western blot (Fig. 1k). Notably, the expression level of Cox-1, a mitochondrially-expressed protein, remained unchanged in MC38 and MC38-SH3 cells. Despite the observed molecular alterations, MCJ-deficiency did not affect in vitro growth and proliferation, suggesting that its functional impact appears to be context-dependent.

### MCJ deficiency enhances mitochondrial respiration and reprograms tumor metabolism

**Fig. 2:**
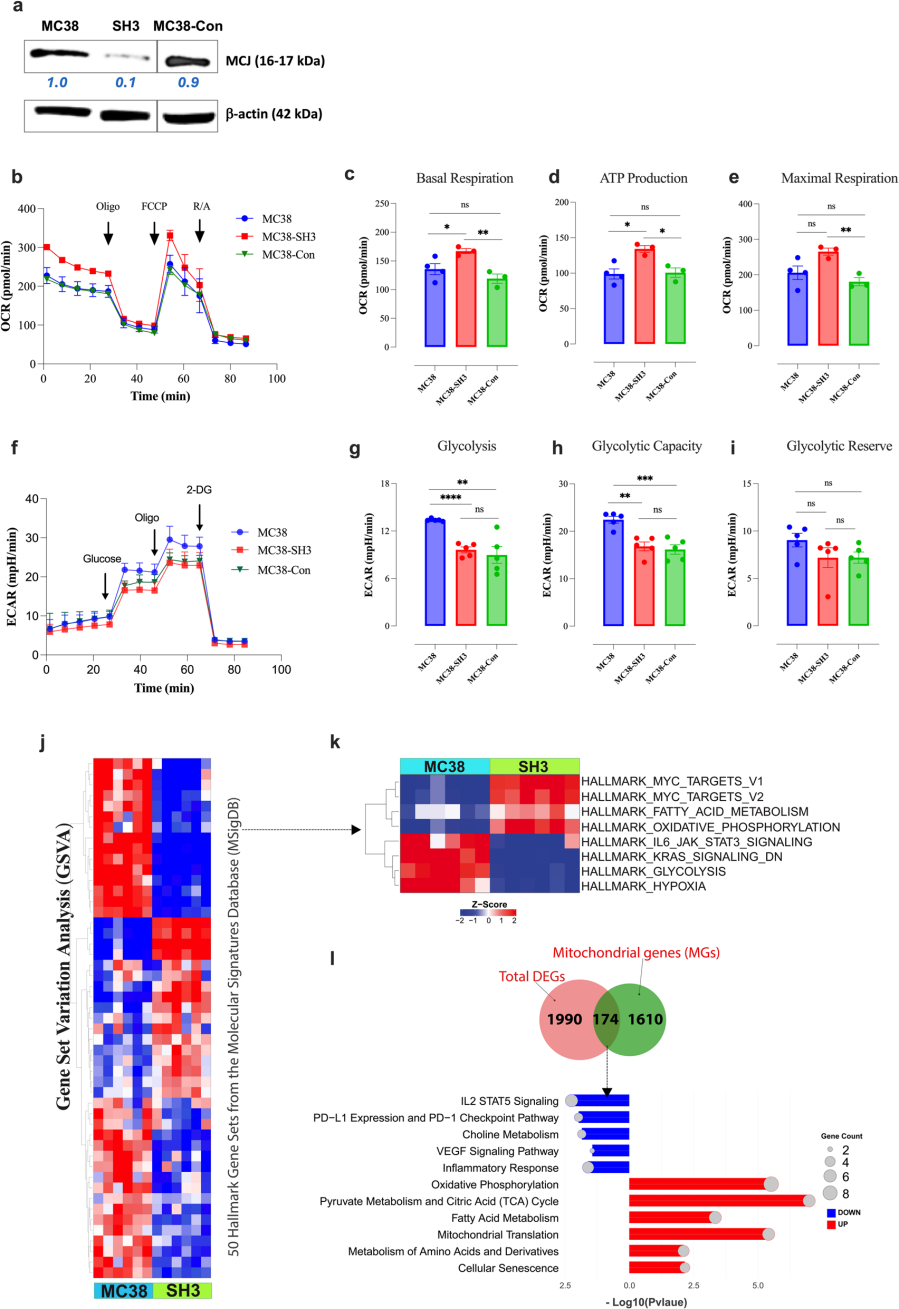
Loss of MCJ increases oxidative phosphorylation-dependent metabolism. **a** Western blot analysis of MCJ expression in parental MC38 cells, MCJ-deficient MC38-SH3 cells, and transfection control cell line, designated MC38-Cont. **b**-**e** Measurement of mitochondrial function using the Seahorse XF Cell Mito Stress Test. **b** Oxygen consumption rate (OCR) of MC38, MC38-SH3, and MC38-Con cells was determined following sequential injections of oligomycin (Oligo), FCCP, and rotenone/antimycin A (R/A). Quantification of basal respiration (**c**), ATP-linked respiration (**d**), and maximal respiration (**e**) derived from OCR measurements. **f**-**i** Measurement of glycolytic pathway using the Seahorse XF Glycolysis Stress Test. **f** Extracellular acidification rate (ECAR) measured following sequential injections of glucose, oligomycin, and 2-deoxy-D-glucose (2-DG). Quantification of glycolysis (**g**), glycolytic capacity (**h**), and glycolytic reserve (**i**) derived from ECAR measurements. Data shown in panels **a-i** are representative of 2–3 experiments each. **j**-**l** Pathway-Specific Gene Set Variation Analysis (GSVA) of metabolic and mitochondrial Genes. **j** A heatmap displaying GSVA enrichment between MC38 and MC38-SH3 tumors using the 50-gene sets from the Molecular Signature Database (MSigDB). **k** The sub-heatmap highlights key metabolic and signaling pathways. **l** Venn diagram showing overlap between total differentially expressed genes (DEGs) and mitochondrial genes, and pathway enrichment analysis of mitochondrial DEGs. Data in all graphs are shown as mean ± SEM. Statistical significance was determined using the unpaired, two-tailed Student's t-test; * *P* ≤ 0.05; ** *P* ≤ 0.01; *** *P* ≤ 0.001; **** *P* ≤ 0.0001; ns, not significant

To determine the functional impact of MCJ deficiency on cellular metabolism, mitochondrial respiration and glycolytic activity were assessed in MC38, MC38-SH3, and control-transfectant (MC38-Con) cells. The MC38-Con cell line has normal levels of MCJ expression (Fig. 2a). Oxygen consumption rate (OCR) measuremenst revealed significantly increased mitochondrial respiration in MC38-SH3 cells, including elevated basal respiration, ATP production and maximal respiratory capacity (Fig. 2b-e). The magnitude and consistency of the observed elevation point to an enhanced mitochondrial oxidative capacity in MCJ-deficient cells. In contrast, glycolytic activity, measured by extracellular acidification rate (ECAR), was significantly reduced in MC38-SH3 cells, with decreases of approximately 25% in glycolysis and 20% in glycolytic capacity relative to parental MC38 cells, while glycolytic reserve was not significantly altered (Fig. 2f-i). These findings indicate a metabolic shift characterized by a reduced reliance on glycolysis and a shift towards mitochondrial oxidative phosphorylation (OXPHOS) in MCJ-deficient cells.

Transciptomic profiling of in vivo grown MC38 and MC38-SH3 tumor tissues corroborated these findings. Gene Set Variation Analysis (GSVA) revealed upregulation of pathways associated with MYC targets, fatty acid metabolism, and OXPHOS, whereas pathways associated with glycolysis, hypoxia and inflammatory signaling were downregulated (Fig. 2j-k; Table S5). Further analysis revealed that among the 2,164 DEGs identified in tumor samples, 174 were mitochondrial genes (Fig. 2l), enriched in pathways related to mitochondrial funcion and energy metabolism, including the tricarboxylic acid (TCA) cycle, pyruvate metabolism, fatty acid metabolism and OXPHOS. Notably, immune-related pathways, such as IL-2/STAT5 signaling, and PD-1/PD-L1 checkpoint regulation, as well as VEGF signaling, which is crucial for vascular development and angiogenesis, were also associated with mitochondrial gene alterations (Fig. 2l; Table S6). These findings highlight a link between metabolism and immune regulation in the TME.

###  MCJ deficiency impairs tumor growth in vivo and is associated with distinct transcriptomic reprogramming

**Fig. 3:**
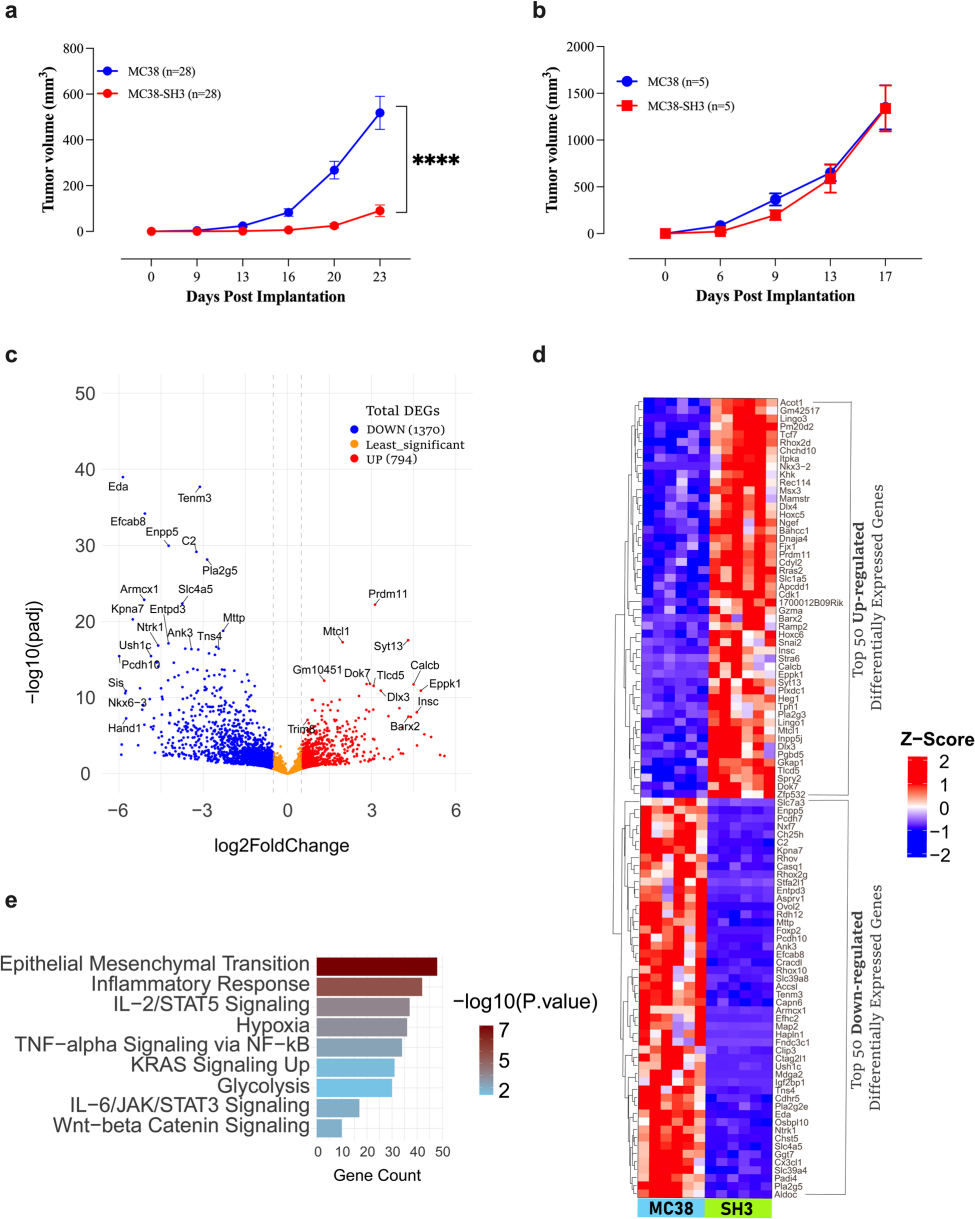
MCJ deficiency impairs tumor growth in immunocompetent hosts and drives distinct transcriptomic reprogramming. Mice were implanted subcutaneously with 1 × 10^5^ MC38 or MC38-SH3 tumor cells. **a** Tumor growth curves of MC38 and MC38-SH3 tumor cells in wild-type C57BL/6 mice. Each data point represents the mean ± SEM of 28 mice per group, pooled from 3 individual experiments. Statistical significance was calculated using 2-way ANOVA. Asterisks denote statistically significant differences between the MC38 and MC38-SH3 groups, **** *P* ≤ 0.0001. **b** Tumor growth curves of MC38 and MC38-SH3 tumor cells in T-cell deficient athymic nude mice. Each point represents the mean ± SEM of 5 mice per group. The data is representative of two independent experiments. **c**-**e** Analysis of RNA-seq data performed on cells from MC38 and MC38-SH3 tumors processed on day 30 post-implantation. **c** Volcano plot depicting DEGs in MC38-SH3 cells. Dotted lines indicate log2 fold change against statistical significance -Log10 (P-adj). Blue dots (left side) signify down-regulated gene expression, red dots (right side) indicate up-regulated gene expression, and orange dots represent genes with the least significant expression differences. **d** Heatmap plot of the top 50 DEGs (both upregulated and downregulated) in MC38-SH3 tumor samples compared to parental MC38. **e** MSigDB over-representation analysis for pathway enrichment. Bar length indicates the total gene count, and color gradient (dark to light) corresponds to the -log10(*p*-value)

In vivo studies using syngeneic C57BL/6 mice revealed that MC38-SH3 tumors had markedly reduced growth, with approximately 71% lower tumor volumes compared to parental MC38 tumors (Fig. 3a). Moreover, there was a significant delay in tumor onset in mice implanted with MC38-SH3 cells (Fig. S1a). At day 13 post implantation, 93% vs. 14% of animals showed palpable MC38 and MC38-SH3 tumors, respectively (Fig. S1a). The substantial difference in growth properties is illustrated by the individual growth curves of MC38 and MC38-SH3 tumor cells (Fig. S1b,c). The fact that MC38-SH3 cells grew at a comparable rate to MC38 cells in vitro but exhibited a dramatically decreased rate in vivo suggested an involvement of extrinsic factors in restricting their growth in syngeneic mice. To investigate the potential involvement of the immune system, we compared the growth of the two cell lines in athymic nude mice. In the absence of T cells, there were no discernible differences in the rates of tumor growth (Fig. 3b and Fig. S1e,f) or incidence (Fig. S1d) between the two cell lines. Restoration of MCJ expression reversed the phenotype, as was demonstrated using a revertant MC38-SH3 cell line (designated SH3-Rev), that re-expressed normal levels of MCJ after being maintained for an extended period of time without selection in G418. This cell line grew indistinguishably from the parental MC38 cells in vivo (Fig. S2), confirming the functional relevance of MCJ expression.

Transcriptomic analysis of excised MC38 and MC38-SH3 tumor tissues revealed 2,164 DEGs (794 upregulated and 1,370 downregulated genes) (Fig. 3c). A heatmap displaying the top 100 upregulated and downregulated genes is shown in Fig. 3d. Functional classification of DEGs revealed their roles in key biological processes as metabolite interconversion and protein-modifying enzymes, gene-specific transcriptional regulators, transporters, and scaffold/adaptor proteins (Fig. S3a), highlighting their involvement in critical cellular processes such as transcriptional regulation, metabolism, and post-translational modifications (Fig. 3e and Fig. S3b). The observed DEGs were enriched in pathways related to EMT, inflammatory cytokine signaling, hypoxia, and oncogenic pathways such as KRAS and Wnt/β-catenin (Fig. 3e). Similarly, over-representation analysis using GO:BP gene sets identified several pathways upregulated in MCJ-deficient tumors, including positive regulation of cell migration and EMT, extracellular matrix organization, regulation of angiogenesis, positive regulation of the inflammatory response and cytokine signaling pathways, and negative regulation of apoptotic process (Fig. S3b). The detailed DEGs and pathway enrichment data are provided in Tables S7-S8. These findings suggest that MCJ deficiency drives extensive molecular reprogramming within the TME.

### MCJ deficiency remodels the tumor immune landscape

**Fig. 4:**
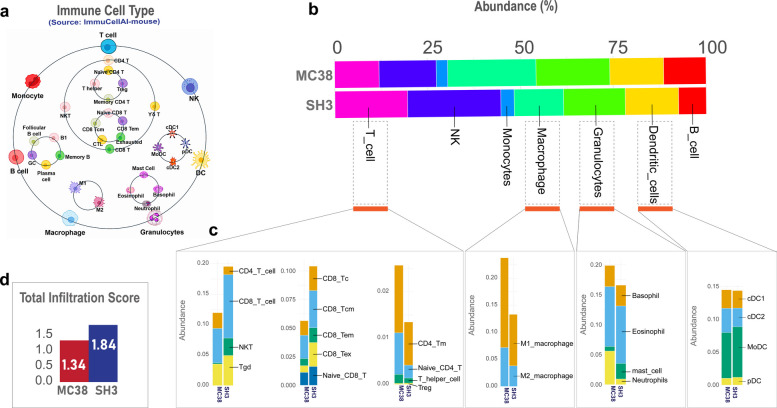
Immune cell abundance analysis: **a** Schematic workflow of the ImmuCellAI-mouse tool, which employs a hierarchical three-layered strategy to estimate the abundance of 36 immune cell subtypes from gene expression data. **b** Comparative estimation of immune cell abundance (%) for seven major immune cell types in parental MC38 and MCJ-deficient MC38-SH3 tumor tisuue samples. **c** Stacked plots depicting the overall absolute abundance of immune cell subtypes in MC38 and MC38-SH3 tumors. **d** Increased overall immune cell infiltration in MC38-SH3 tumors compared to parental MC38, as indicated by the abundance scores

Immune deconvolution analysis was performed using Immune Cell Abundance Identifier for Mouse (ImmuCellAI-mouse) to predict immune cell abundance based on specific transcriptomic signatures. The analysis revealed increased overall immune infiltration in MCJ-deficient tumors (Fig. 4a-d). Notably, there was an enrichment of anti-tumor immune populations, including CD8^+^ T cells, NK cells, and dendritic cells, alongside a reduction in pro-tumor populations such as granulocytes and macrophages (Fig. 4b). Subpopulation analysis demonstrated increased abundance of CD8^+^ T cells, including naïve, effector memory, and exhausted cells (Fig. 4c). Among myeloid cells, a reduction in both M1 and M2 macrophages was noted, while the decline in granulocytes was largely driven by a marked decrease in neutrophils. The abundance score revealed an increase in the total infiltration of immune cells,, indicative of enhanced immune activation within MCJ-deficient tumors (Fig. 4d; Table S9).

### Enhanced cytotoxic immune responses in MCJ-deficient tumors

**Fig. 5:**
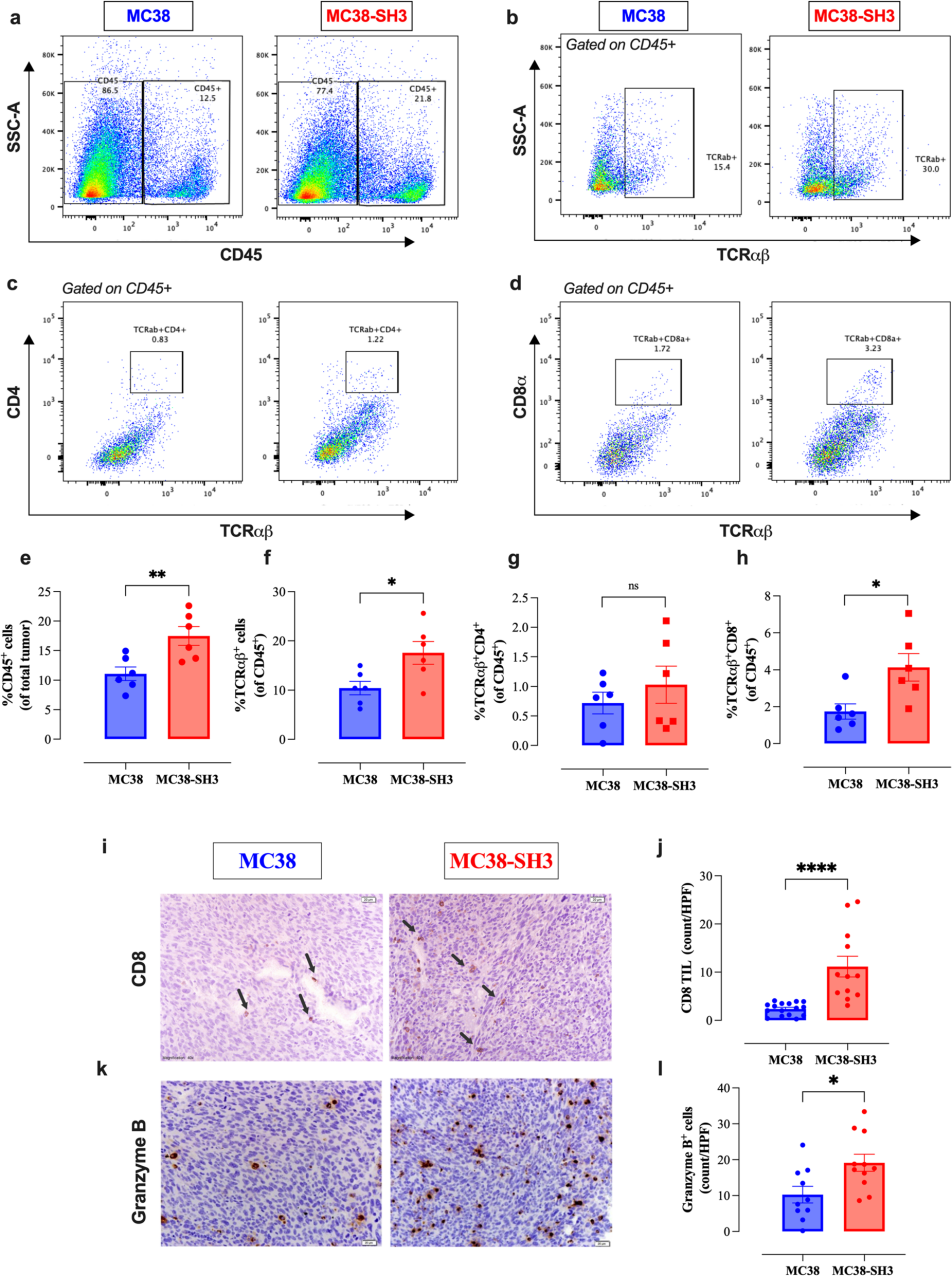
MCJ deficiency enhances T cell infiltration and functionality in tumors. The percentages of intratumoral immune cells were determined by flow cytometry (panels **a**-**h**) and immunohistochemistry (panels **i**-**l**). Representative dot plots and quantification of the percentages of CD45^+^ immune cells (**a**, **e**), total TCRab^+^ cells (**b**, **f**), TCRαβ^+^CD4^+^ T cells (**c**, **g**) and TCRαβ^+^CD8.^+^ T cells (**d**, **h**). Representative images of Immunohistochemical staining and quantification of CD8⁺ T cells (**i**, **j**), and granzyme B⁺ cells (**k**, **l**) per high-power field in MCJ-deficient tumors. Immunohistochemical staining was performed at 40 × magnification (scale bar: 20 µm). Each data point in the bar graphs represents a single mouse, pooled from two independent experiments. Statistical significance was calculated using the unpaired Student’s t-test. Asterisks denote statistically significant differences between the MC38 and MC38-SH3 groups, * *P* ≤ 0.05; ** *P* ≤ 0.01; **** *P* ≤ 0.0001; ns, not significant, *p ≥* 0.05

Next, we utilized multi-color flow cytometry to assess the extent of tumor-infiltrating immune cell populations. The gating strategy used to identify the lymphoid subpopulations is shown in Fig. S4. There was a significant increase in the proportions of CD45^+^ leukocytes, TCRαβ^+^ lymphocytes, and CD8^+^ T cells in MC38-SH3 tumors (Fig. 5a-h). IHC staining further demonstrated a 4.7-fold increase in CD8^+^ T cells and a ~ twofold increase in granzyme B (GZMB)-positive cells in MC38-SH3 tumors, suggesting enhanced functionality of these cytotoxic cells against tumor tissue (Fig. 5i-l). IHC analysis also showed that the absolute number of GZMB-positive cells per high power field was higher than that of CD8^+^ T cells (mean ± SEM: 19.1 ± 2.4 vs. 11.2 ± 2.1, *p* = 0.02), suggesting the presence of additional cytotoxic cell types in MCJ-deficient tumors. These findings indicate enhanced cytotoxic immune activity, likely mediated by both CD8^+^ T cells and NK cells, contributing to growth suppression of MCJ-deficient tumors.

### MCJ-deficient tumors display alterations in myeloid cell populations

**Fig. 6:**
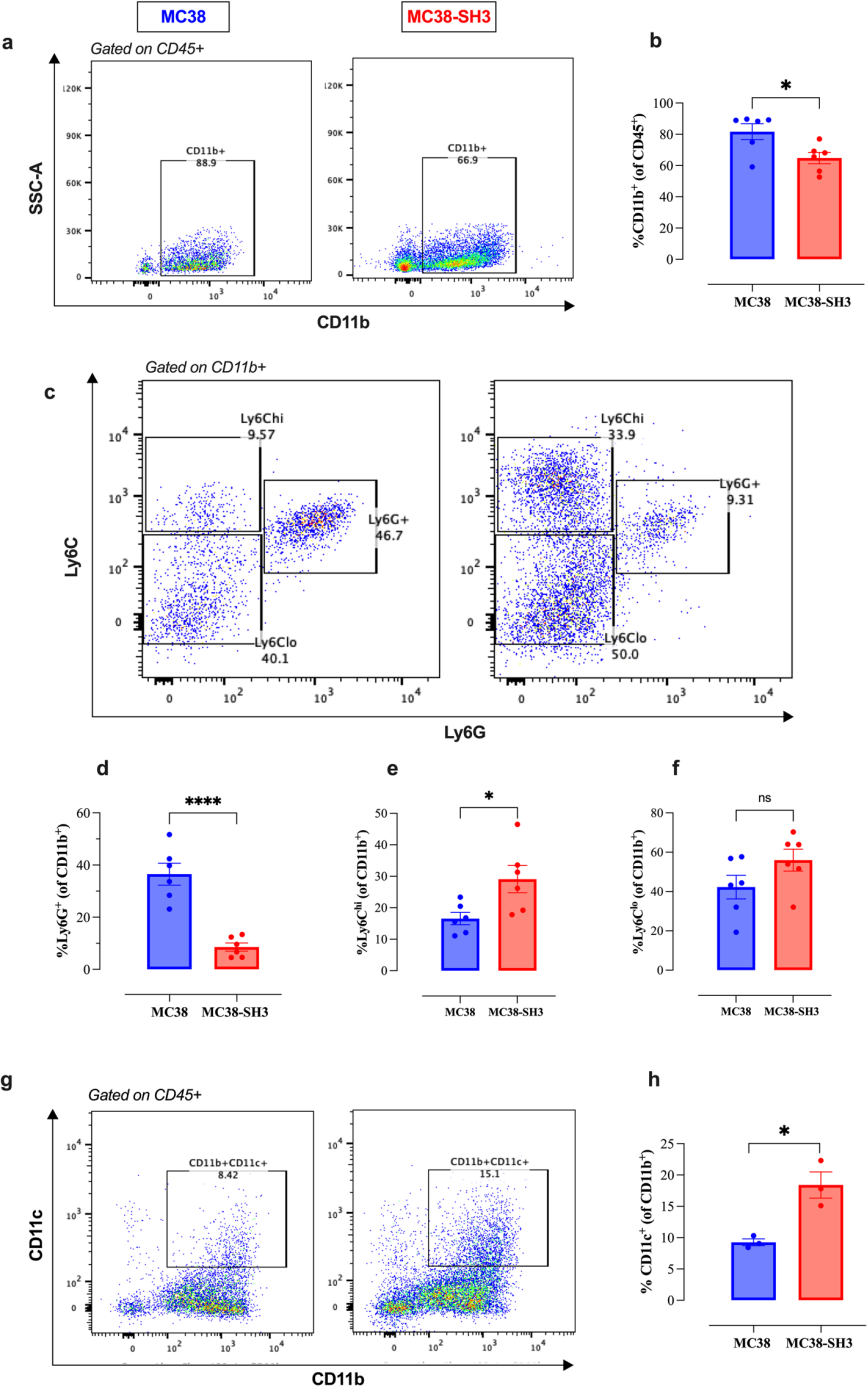
MCJ deficiency alters the composition of the myeloid subpopulations within the tumor microenvironment. The percentages of intratumoral myeloid cells were determined by flow cytometry. Representative dot plots and the quantification of the percentages of CD11b^+^ myeloid cells (**a**, **b**), Ly6G^+^ granulocytes (**c**, **d**), Ly6C^hi^ monocytes (**c**, **e**), Ly6C^lo^ macrophages (**c**, **f**) and CD11b^+^CD11c.^+^ dendritic cells (**g**, **h**). Each data point represents a single mouse, pooled from two independent experiments. Statistical significance was calculated using the unpaired Student’s t-test. Asterisks denote statistically significant differences between the MC38 and MC38-SH3 groups, * *P* ≤ 0.05; **** *P* ≤ 0.0001; ns, not significant, *p ≥* 0.05

The intratumoral myeloid cell compartment was analyzed by flow cytometry (refer to Fig. S5 for the gating strategy). MCJ-deficient tumors exhibited a marked reduction in the percentage of CD11b^+^ myeloid cells, primarily due to a significant (4.3-fold) reduction in Ly6G^+^ granulocytic cells (36.5% in MC38 vs. 8.5% in MC38-SH3 tumors; *p <* 0.0001) (Fig. 6a-d), which likely represent granulocytic myeloid-derived suppressor cells [15]. Conversely, Ly6C^hi^ and Ly6C^lo^ monocytic cell populations were increased (Fig. 6c, e, f), along with CD11c^+^ dendritic cells (Fig. 6g, h), indicating a shift toward a less immunosuppressive and more immunostimulatory TME in MC38-SH3 tumors.

### Enhanced MHC class I expression on MCJ-deficient tumor cells

**Fig. 7:**
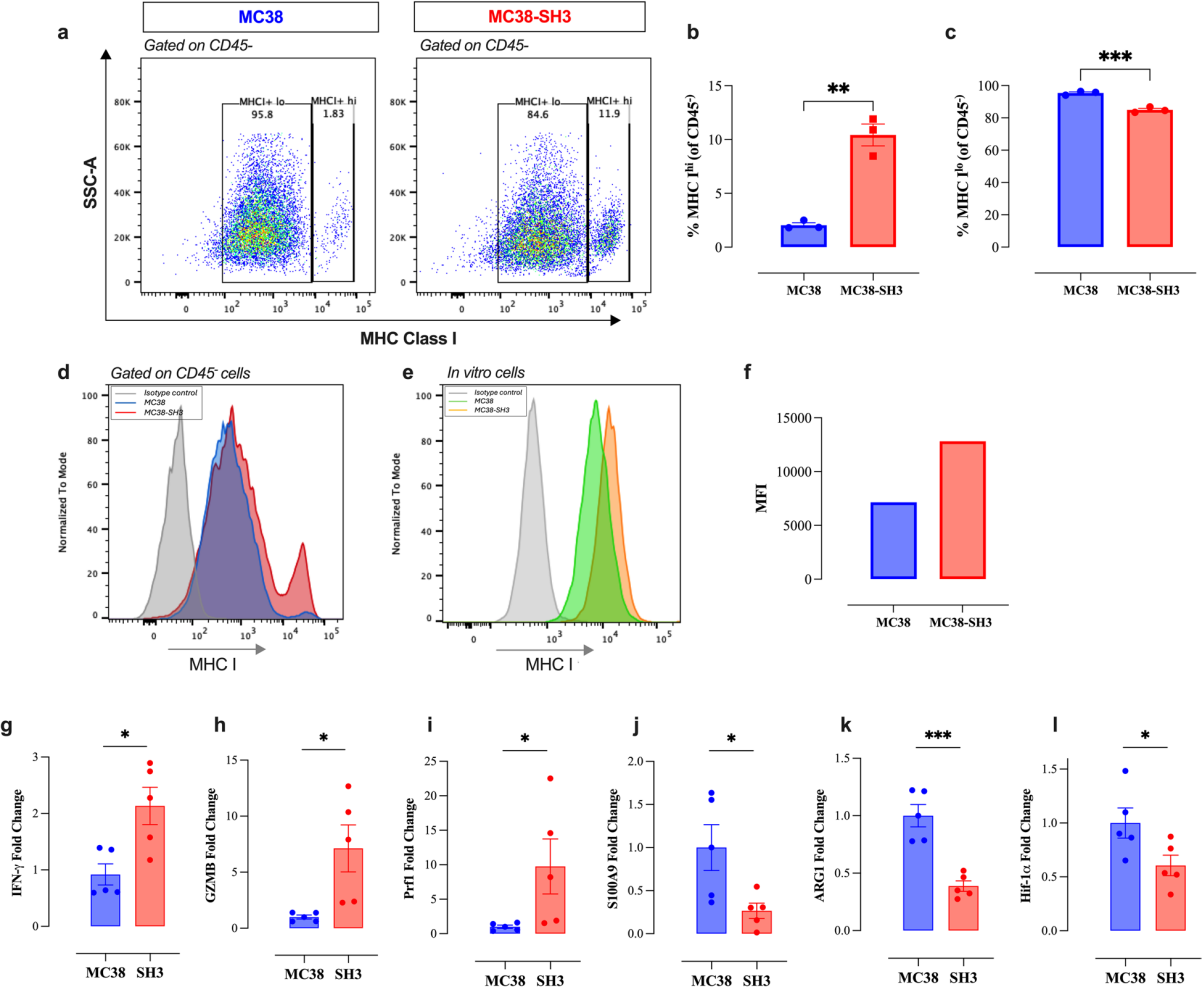
Enhanced immunogenicity of MCJ-deficient tumors. The expression of MHC class I on CD45^−^ tumor-associated cells was assessed by flow cytometry. Representative dot plots and the quantification of the percentages of MHC-I^hi^ (**a**, **b**), and MHC-I^lo^ (**a**, **c**) cells in MC38 and MC38-SH3 tumors. **d** Representative overlay histograms showing MHC-I expression on CD45^−^ cells in MC38 and MC38-SH3 groups. The grey histogram indicates staining with isotype control. **e** Representative overlay histograms showing MHC-I expression on in vitro grown MC38 and MC38-SH3 groups. The grey histogram indicates staining with isotype control. **f** Median fluorescence intensity of MHC-I on in vitro grown MC38 and MC38-SH3 cells. RNA was extracted from total MC38 and MC38-SH3 tumor tissues and gene expression levels were determined using qRT-PCR. The effect of MCJ deficiency on the expression levels of **g** IFN-γ, **h** GZMB, **i** Prf1, **j** S100A9, **k** ARG1 and **l** HIF-1α was assessed. The bar graph represents the mean ± SEM for each marker expressed as fold change relative to MC38. Statistical significance was calculated using the unpaired Student’s t-test. Asterisks denote statistically significant differences between the MC38 and MC38-SH3 groups, * *P* ≤ 0.05; ** *P* ≤ 0.01; *** *P* ≤ 0.001; ns, not significant, *p ≥* 0.05

Antigen presentation by MHC class I is crucial for CD8^+^ T cell-mediated recognition and killing of tumor cells. We therefore investigated whether MCJ deficiency could influence the expression of MHC class I proteins on tumor cells in the TME. Flow cytometry analysis revealed that the majority of MC38 tumor cells displayed low or intermediate levels of MHC class I, while a small percentage exhibited high levels of MHC class I protein (Fig. 7a-d). However, a significant fivefold increase was observed in the proportion of MCJ-deficient tumor cells expressing high levels of MHC class I (Fig. 7a, b). Analysis of MHC class I expression on in vitro grown tumor cells demonstrated a similar increase in MHC class I expression on MC38-SH3 cells (Fig. 7e, f). This enhancement likely improves antigen presentation and recognition by CD8^+^ T cells. This conclusion is supported by changes in key cytokines/effector molecules detected by RT-PCR in tumor tissues (Fig. 7g-l), demonstrating a significant increase in the expression of IFN-γ, granzyme B and perforin in MC38-SH3 tumors (Fig. 7g-i). Conversely, a significant reduction in the expression of S100A9, arginase-1 and HIF-1α markers was observed in MC38-SH3 tumors (Fig. 7j-l). Taken together, these findings highlight the fundamental alterations in MC38-SH3 tumors that underlie their increased immunogenicity and reduced proliferation in vivo.

### Clinical relevance of MCJ (DNAJC15) expression in human colorectal cancer

**Fig. 8:**
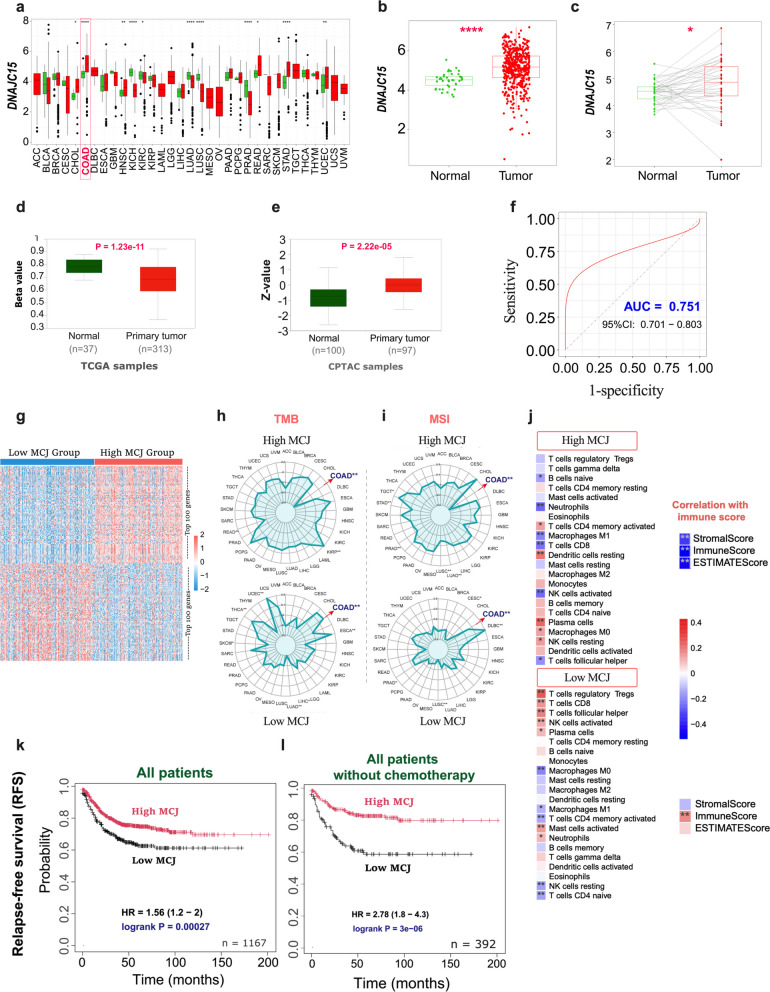
Clinical, molecular, and prognostic associations of MCJ (DNAJC15) expression in colorectal adenocarcinoma (COAD).** a** Pan-cancer analysis of *DNAJC15* expression across 33 tumor types (TCGA) based on unpaired normal and tumor samples, with COAD highlighted. Statistical significance is indicated; * *P* ≤ 0.05; ** *P* ≤ 0.01; *** *P* ≤ 0.001; **** *P* ≤ 0.0001; ns, not significant. **b** Box plots showing MCJ expression levels between unpaired normal and COAD tumor samples. **c** Box plots of paired analyses of MCJ expression between matched normal and tumor samples from the same patients. **d** Box plot illustrating promoter methylation levels (beta values) in normal and primary COAD tumor samples from the TCGA. **e** Box plot showing MCJ protein levels in normal and primary COAD samples from Clinical Proteomic Tumor Analysis Consortium (CPTAC) datasets. **f** Receiver operating characteristic (ROC) curve evaluating the ability of MCJ expression to discriminate COAD tumor from normal tissue. **g** Heatmap of the top 100 DEGs between high- and low-MCJ expressing COAD tumors. **h** Radar plot demonstrating the correlation between high- and low-MCJ expression groups with tumor mutational burden (TMB). The high-MCJ group shows a significant negative correlation with TMB in COAD, whereas the low-MCJ group shows a significant positive correlation with TMB in COAD and other cancers. **i** Radar plot showing the association between MCJ expression groups and microsatellite instability (MSI). The high-MCJ group is negatively correlated with MSI in COAD, while the low-MCJ group is positively correlated with MSI in COAD. Significance levels: *p < 0.05, **p < 0.01. **j** Heatmap depicting correlations between high- and low-MCJ expressing COA tumors, immune cell infiltration ratios and immune scores. **k**-**l** Kaplan–Meier (KM) survival plots depicting the association between high- or low-expressing COAD with relapse-free survival (RFS) in all patients (**k**) or in patients who did not receive chemotherapy (**l**)

The clinical relevance of MCJ expression was investigated across multiple cancer datasets, with a particular focus on colorectal adenocarcinoma (COAD). Pan-cancer analysis revealed variable DNAJC15 gene expression across tumor types, with COAD highlighted as one of the cancers with significantly elevated expression relative to normal tissue (Fig. 8a, b). Paired analysis of matched normal-tumor samples further confirmed tumor-associated increase in DNAJC15 expression (Fig. 8c), which was associated with promoter hypomethylation (Fig. 8d). Consistent with this finding, proteomic analysis demonstrated significantly increased MCJ protein abundance in primary tumors (Fig. 8e). Furthrmore, diagnostic reciever operating characterstic (ROC) analysis demonstrated that the expression of MCJ reliably differentiates tumor from normal tissues (AUC = 0.751; Fig. 8f).

We next assessed the relationship between MCJ expression and immune-related features. Transcriptomic profiling comparing high/low-expressing MCJ tumors revealed broad differences in gene expression patterns (Fig. 8g). Stratification by MCJ expression further showed associations with tumor mutational burden (TMB) and microsatellite instability (MSI) across cancers (Fig. 8h, i). Compared with other cancer types, COAD exhibited distinct MCJ-dependent association patterns, with low MCJ-expression correlating with high TMB and MSI (Fig. 8h, i). MCJ expression was also associated with distinct immune cell infiltration patterns (Fig. 8j), with low expression correlating with a significant rise in the abundance of several immune cell populations (such as CD8 T cells, Treg cells, T_FH_ cells and activated NK cells), and showing strong associations with stromal, immune and ESTIMATE scores (Fig. 8j). Furthermore, patients with high MCJ-expressing COAD exhibited significantly improved survival compared with patients whose tumors expressed relatively low MCJ protein (Fig. 8k). This association was further strengthened in the subgroup analysis of COAD patients without chemotherapy, where high MCJ expression remained linked to markedly better survival (Fig. 8l). Together, these results indicate that elevated MCJ expression is associated with improved clinical outcome.

## Discussion

Metabolic reprogramming, a critical hallmark of cancer, involves a complex interplay of molecular pathways that facilitates the uncontrolled proliferation and survival of malignant cells [16]. Tumor cells exhibit remarkable adaptability, modifying their metabolic profiles in response to environmental factors such as nutrient availability, hypoxia and immune system pressure. Even in the presence of adequate oxygen, however, tumor cells preferentially utilize glycolysis over OXPHOS, a phenomenon known as the Warburg effect [17, 18]. This shift enables malignant cells to meet their bioenergetic demands and accumulate metabolic intermediates necessary for rapid proliferation. Despite extensive research on the Warburg effect and its potential for therapeutic targeting, much of the underlying mechanisms remains unknown. As an endogenous inhibitor of complex I of the ETC, MCJ has emerged as a key regulator of mitochondrial respiration [5]. The findings of the current study demonstrate that MCJ deficiency leads to a profound alteration in tumor metabolic activity, shifting from utilizing the glycolytic pathway to OXPHOS-dependent metabolism. This was accompanied by enhanced immunogenicity and reduced tumor growth in vivo, effectively transforming “cold” tumors to “hot” tumors [14].

MCJ deficiency led to an upregulation of the OXPHOS pathway, which was associated with alterations in the level of mRNA and protein of various complex 1 subunits. While the majority of the subunits were upregulated, a subset exhibited decreased expression, perhaps reflecting a fine-tuned balance in the ETC to prevent excess ROS production as a result of electron leakage [19]. Analysis of the other ETC complexes revealed an upregulation in key subunits of complexes III and IV, suggesting a potential enhancement in mitochondrial respiration and OXPHOS activity, which is in line with previous studies [19, 20]. Moreover, transcriptomic profiling of MCJ-deficient cells revealed an enhancement in EMT as a consequence of the increased OXPHOS activity. This observation is similar to previous findings linking OXPHOS activity to enhanced EMT and metastatic potential in MDA-MB-468 and MCF-7 breast cancer cell lines [21].

Investigation of the in vivo growth dynamics of MCJ-deficient tumor cells indicated a significantly slower growth rate in syngeneic mice, compared to the parental MC38 tumors. Importantly, the growth of parental and MCJ-deficient MC38 cells was identical in athymic nude mice. Our findings are in line with a recent paper that reported similar growth characteristics of MCJ-deficient melanoma cells in immunocompetent mice [22].

The TME is a complex and continuously evolving ecosystem that dictates whether tumor growth is held in check or allowed to progress and metastasize [23]. Based on flow cytometric findings, as well as immune deconvolution analysis using ImmuCellAI-mouse [24], MCJ deficiency induces alterations in the cellular constituents within the TME leading to reduced tumor growth. This is manifested by a significant increase in the percentage of infiltrating CD45^+^ leukocytes in MC38-SH3 tumors, particularly in both CD8^+^ T cells and NK cells, the two main cytotoxic cell populations that act to limit tumor growth [25]. Immunohistochemical analysis confirmed the increased abundance of CD8^+^ T cells in MC38-SH3 tumors and further revealed a corresponding increase in granzyme B-positive cytotoxic cells. Thus, the current study provides insight into the potential mechanism underlying the observed reduction in tumor growth, revealing an augmentation in the percentage of tumor-infiltrating CD8^+^ T lymphocytes and NK cells and a concurrent enhancement in their activation status [26].

The metabolic rewiring induced by the loss of MCJ was associated with increased MHC class I expression on tumor cells. A subset of tumor cells that exhibited > tenfold higher levels of MHC class I expression was significantly enriched (> fivefold) in MC38-SH3 tumors compared to MC38 cells, which enhances antigen presentation and recognition by cytotoxic CD8^+^ T cells, thereby contributing to increased tumor immunogenicity. This finding is consistent with a recent study that also demonstrated increased MHC class I expression in MCJ-deficient melanoma cells, driven by enhanced complex I activity that induces the accumulation of succinate, promoting the transcriptional activation of antigen-presentation genes, ultimately increasing MHC class I surface expression, tumor immunogenicity and cytotoxic T cell activation [22]. Moreover, the link between MCJ deficiency and increased MHC class I expression on tumor cells may be mediated through changes in hypoxic conditions. We have demonstrated that, in both MC38 murine tumors and human colorectal cancers, lower MCJ expression correlates with decreased hypoxia. Hypoxia has been shown to promote tumor evasion by reducing MHC class I expression on tumor cells [27]. Moreover, there is evidence that metabolic rewiring of tumor cells to utilize OXPHOS leads to an upregulation of MHC class I expression through the ERK5 MAPK signaling pathway [28]. Therefore, it is not unreasonable to propose that the induced upregulation in the expression of MHC class I proteins may be mediated via a reduction in hypoxic conditions within the TME and the activation of ERK5 MAPK pathway.

Our study also revealed an association between poor in vivo growth of MCJ-deficient tumors and the composition of the myeloid populations within the TME, including tumor-associated macrophages (TAMs), MDSCs, tumor-associated neutrophils (TANs), and DCs [29]. In the context of TME, myeloid cells have dual functions, with some subsets contributing to anti-tumor immune responses and tumor growth inhibition, while other subsets acting to maintain the immunosuppressive conditions and promote tumor growth [30, 31]. Flow cytometry analysis revealed a significant decrease in the overall percentage of intratumoral CD11b^+^ myeloid cells, primarily due to the loss of Ly6G^+^ granulocytes, in MCJ-deficient tumors. This observation also correlated with a marked reduction in the expression of S100A9 and arginase-1, known biomarkers of myeloid-derived suppressor cells [32, 33]. In contrast, the other two myeloid cell subsets, namely CD11b^+^Ly6C^hi^ Ly6G^−^ and CD11b^+^Ly6C^low^ Ly6G^−^ cells, were increased in MC38-SH3 compared to MC38 tumors (13.6% and 34.5% vs. 18.8% and 36.3%, respectively). The change in myeloid cell function is confirmed by the significant reduction in arginase 1 expression in MCJ-deficient tumors. This enzyme is considered a biomarker of immunosuppressive MDSCs and plays a significant role in promoting tumor evasion through depletion of L-arginine and inhibiting anti-tumor T cell response [34, 35].

Transcriptomic profiling of tumor tissue revealed significant alterations in a large number of genes which collectively affect important biological processes, including ATP metabolism, cell proliferation and differentiation, cell migration and invasion, EMT, angiogenesis, inflammatory and immune responses, hypoxia, apoptosis, and signal transduction pathways. Moreover, GSVA analysis revealed pathway activity changes in MC38-SH3 tumors associated with increased MYC activation, fatty acid metabolism, and OXPHOS, which play critical roles in the regulation of cancer progression [36–38]. The concurrent activation of fatty acid metabolism and OXPHOS pathways in MC38-SH3 tumors suggest inter- dependence on fatty acids for increased energy production needed for rapid growth. There is evidence linking fatty acid metabolism-related enzymes with the promotion of metastasis in colorectal cancer through the regulation of angiogenesis and EMT [36]. On the other hand, some key metabolic and signaling pathways were downregulated in MC38-SH3 tumors, including glycolysis and hypoxia. As mentioned earlier, tumor cells undergo metabolic reprogramming and use the glycolytic pathway to meet their energy demands [39]. It appears that the absence of MCJ re-programs tumor cells so that they become dependent on OXPHOS to generate ATP. The reduced HIF-1α expression in MC38-SH3 tumors is in line with the reduction in hypoxia and glycolysis pathways. HIF-1α is stabilized under the hypoxic tumor conditions and plays a key role in the regulation of cancer progression [40]. Taken together, this data highlights the fundamental shift from a relatively immunosuppressive TME in MC38 tumors to the more permissive immunogenic environment in MC38-SH3 tumors.

It is important to emphasize that the findings reported in MCJ-deficient tumor cells are consistent with our analysis of human colorectal cancers. This analysis indicated that MCJ expression is consistently elevated in colorectal tumors through epigenetic and proteomic regulation compared to normal tissue. Importantly, MCJ expression stratified patient tumors into biologically distinct subgroups characterized by differences in TMB, MSI, and immune and stromal composition, features that are well established to influence tumor progression and therapeutic responsiveness [41, 42]. The observed increase in TMB and MSI in low MCJ-expressing tumors could also underlie the increased immunogenicity in human colorectal cancers. Nevertheless, it is intriguing to observe that despite the increased immunogenicity observed in tumors expressing low MCJ, relapse-free survival was significantly poorer in colorectal cancer patients, suggesting a more complex MCJ-associated tumor biology. These findings suggest that MCJ expression may regulate other critical functions of cancer cells, such as EMT transition or metastatic potential [43].

The current study provides strong evidence for a role for MCJ in regulating tumor metabolism and immunogenicity. Nevertheless, several limitations should be highlighted. First, the findings rely primarily on a single murine colorectal cancer cell line (MC38), which may limit generalizability to other tumor types or human systems. Although human dataset analyses were included, they are largely correlative in nature and do not necessarily establish causality. Second, the study utilizes siRNA-mediated knockdown rather than genetic knockout, for example by CRISPR-Cas9, raising the possibility of residual MCJ activity. Third, while immune involvement is strongly implicated, the mechanistic links between metabolic reprogramming and specific immune responses remain incompletely defined. Additionally, the in vivo experiments are confined to mouse models, which may not fully recapitulate the complexity of human cancers. Fourth, cell lines in which MCJ is over-expressed should also be investigated. Such studies would be valuable to our understanding of the effect of metabolic rewiring in tumors. Finally, the apparent contradiction between increased immunogenicity in MCJ-deficient tumors and poorer survival in human patients with low MCJ expression highlights unresolved complexities that warrant further investigation.

In conclusion, our findings highlight the role of MCJ in regulating the bioenergetic landscape in the TME through the rewiring of the ETC, shifting the predominantly immunosuppressive hypoxic conditions to a more metabolically active, inflammatory, milieu. The approach of targeting MCJ protein to modulate the metabolic environment within the TME could be exploited to boost antitumor immunity. There are numerous ongoing clinical trials investigating agents that target different mitochondrial proteins [44]. This underscores the potential utility of targeting mitochondrial metabolism for cancer therapy.

## Materials and methods

A detailed description of the Materials and Methods used in this study is provided in the Supplementary M&M.

### Generation of MCJ-deficient cell lines

The murine colon adenocarcinoma MC38 cell line was maintained as previously described [45]. MCJ-deficient transfectants were derived using an MCJ siRNA plasmid obtained from Dr. Mercedes Rincon, as described [5]. Full details are given in the Supplementary M&M. An MCJ-deficient cell line, derived as a stable transfectant of MC38 cells, was designated MC38-SH3 and used for all subsequent experiments (described in detail in the Results section). A cell line, designated MC38-Con, which had undergone the same transfection procedure and retained parental MCJ expression, was used as control in some studies. Additionally, a revertant cell line of MC38-SH3 cells was derived after long-term culture (4–6 weeks) of the cells without G418 selection. This cell line was designated SH3-Revertant (or SH3-Rev).

### Western blot analysis

This was carried out using a standard protocol in our laboratory, as described [46]. Additional details are provided in the Supplementary M&M.

### Cell proliferation assay

The impact of MCJ knockdown on cellular proliferation was assessed as described in the Supplementary M&M.

### Mitochondrial and glycolytic stress tests

Oxygen consumption rate (OCR) and extracellular acidification rate (ECAR) were measured using the Seahorse XF Mito Stress Test and Glycolytic Stress Test kits (Agilent), respectively, following manufacturer’s instructions (refer to the Supplementary M&M for details).

### Experimental animals

The experimental animals used in this study, C57BL/6 mice and athymic NMRI/nude^nu/nu^ mice, have been described [47]. All animal studies were performed in accordance with and after approval of the Animal Research Ethics Committee of the United Arab Emirates University (Protocols #ERA_2018_5743 and ERA_2024_4434).

### In vivo tumor studies

Tumor implantation and growth studies were carried out essentially as previously detailed [47, 48]. Full description is provided in the Supplementary M&M.

### Flow cytometry

Cellular alterations in the tumor immune microenvironment were analyzed by multi-color flow cytometry, as detailed previously [45]. Refer to the Supplementary M&M for complete details.

### Immunohistochemistry (IHC) staining

Tumor sections were analyzed by IHC for CD8 + and granzyme B-positive cells as per established protocols in our laboratory [4, 48]; complete details are given in the Supplementary M&M.

### Quantitative real-time PCR

The level of gene expression within the tumor tissue of key immune biomarkers, including IFN-γ, Perforin-1, Granzyme B, S100A9, HIF1α, and Arginase 1, was analyzed by qRT-PCR, as described [45, 47]. Details are provided in the Supplementary M&M.

### Whole transcriptome sequencing and bioinformatics analyses of tumors

Bulk RNASeq was performed on RNA extracted from in vitro cultured MC38 and MC38-SH3 transfectant cells as well as from single-cell suspensions of in vivo grown tumor tissues. Complete details of the RNA extraction and sequencing, preprocessing of RNASeq data for quality control, differential expression analysis, functional and pathway enrichment analysis of DEGs, and immune deconvolution analysis are provided in the Supplementary M&M.

### Correlation analysis of MCJ (DNAJC15) expression in human cancers

To provide a clinical context, we analyzed the relative expression of DNAJC15 in human tumors, with a special focus on colon adenocarcinoma (COAD) in comparison to normal tissue, and correlated the level of expression with tumor mutation burden (TMB) and microsatellite instability (MSI). Furthermore, the relationship between the level of DNAJC15 expression in human COAD and immune-related genes was assessed and correlated with the recurrence-free survival (RFS) in patients. analysis. Details on the methodology used for this analysis is provided in the Supplementary M&M.

### Statistical analysis

Statistical significance between experimental groups was analyzed using 2-way ANOVA or the unpaired, two-tailed Student's t-test, as indicated. All analysis was done using the statistical program of GraphPad Prism software version 10 (San Diego, CA). Statistical significance was shown as * (*P* ≤ 0.05), ** (*P* ≤ 0.01), *** (*P* ≤ 0.001) and **** (*P* ≤ 0.0001).

## Supplementary Information

Supplementary Material 1.

Supplementary Material 2.

## Data Availability

All data generated or analyzed during this study are included in the published article and its supplementary information files.
