# Supplementary material for: Modulation of the immunogenic landscape in colorectal cancer by mitochondrial methylation-controlled J protein
Source: Mol Biomed. 2026 May 11;7:67. doi: 10.1186/s43556-026-00466-9 (PMC13161452; doi:10.1186/s43556-026-00466-9)
Supplement: Supplementary file 1 — Supplementary Material 1. [file 43556_2026_466_MOESM1_ESM.docx]

**Modulation of the immunogenic landscape in colorectal cancer by mitochondrial Methylation-Controlled J protein**

Maram Ahmed, Shoja M. Haneefa, Aftab Alam, *et al.*

**Supplementary Figures**

**
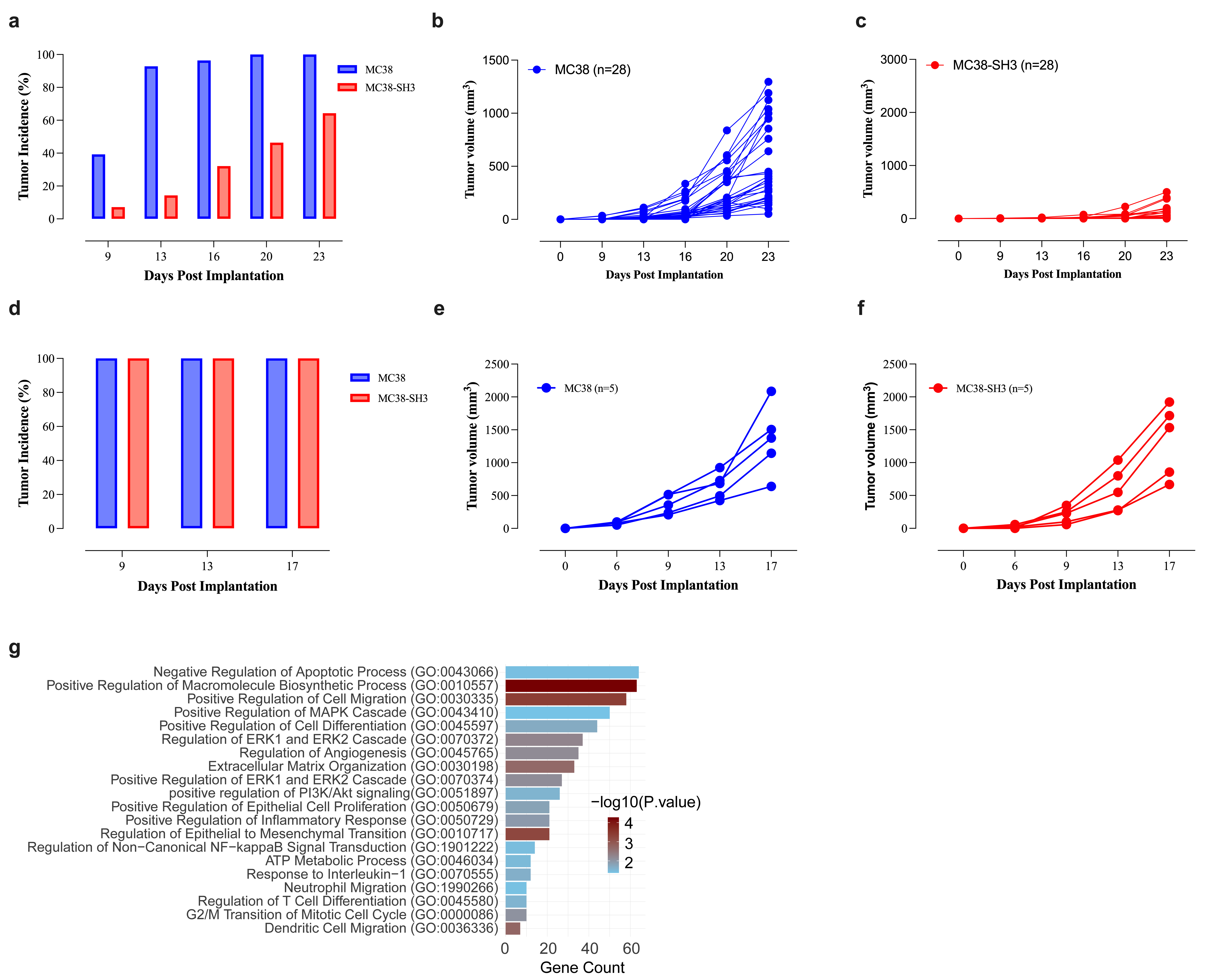
**

**Figure S1. MCJ deficiency impairs tumor growth in immunocompetent but not immunocompromised hosts.** Mice were implanted subcutaneously with (1x10^5^) MC38 or (1x10^5^) MC38-SH3 tumor cells. (**a**) Incidence and individual growth curves of (**b**) MC38 and (**c**) MC38-SH3 tumor cells in wild-type C57BL/6 mice. The data is representative of three individual experiments. Nude mice were implanted subcutaneously with (2x10^6^) MC38 or MC38-SH3. (**d**) Incidence and individual growth curves of (**e**) MC38 and (**f**) MC38-SH3 tumor cells in T-cell deficient athymic nude mice. The data is representative of two independent experiments.

**
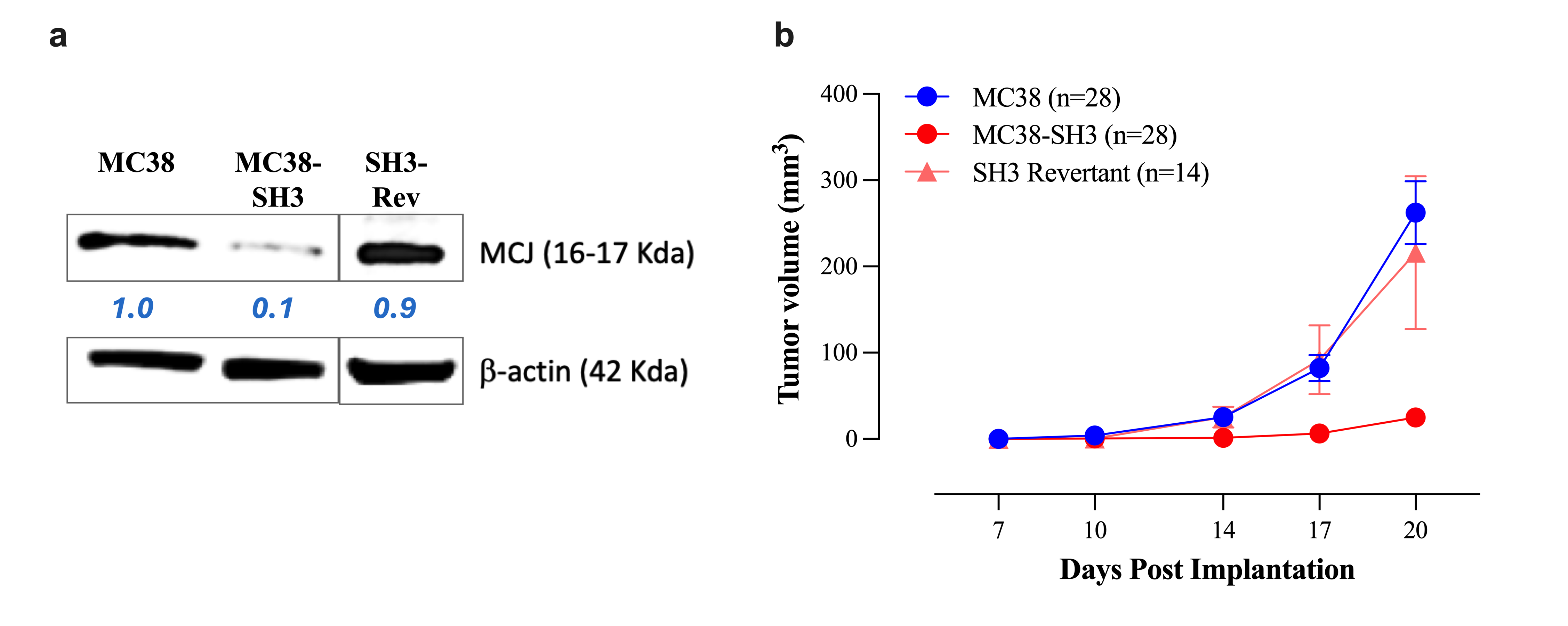
**

**Figure S2. Restoration of MCJ expression in MC38-SH3 cells normalizes their ability to grow in syngeneic mice.** A revertant of MC38-SH3 cells was derived by growing the cells in the absence of G418 selection. (**a**) Western blot analysis of MCJ expression in parental MC38 cells, MCJ-deficient MC38-SH3 cells, and SH3-Rev cells. The SH3-Rev cell line expresses normal levels of MCJ protein levels. (**b**) Mice were implanted s.c. with 1x10^5^ MC38, MC38-SH3, or SH3-Rev tumor cells; tumor growth was then followed for 3-4 weeks. Each data point represents the mean ± SEM of 28 mice per group (pooled from 3 experiments) for MC38 and MC38-SH3 cells or 14 mice pooled from 2 experiments (for SH3-Rev cells). The data for MC38 and MC38-SH3 cells are as shown in Figure 3 of the manuscript.

**
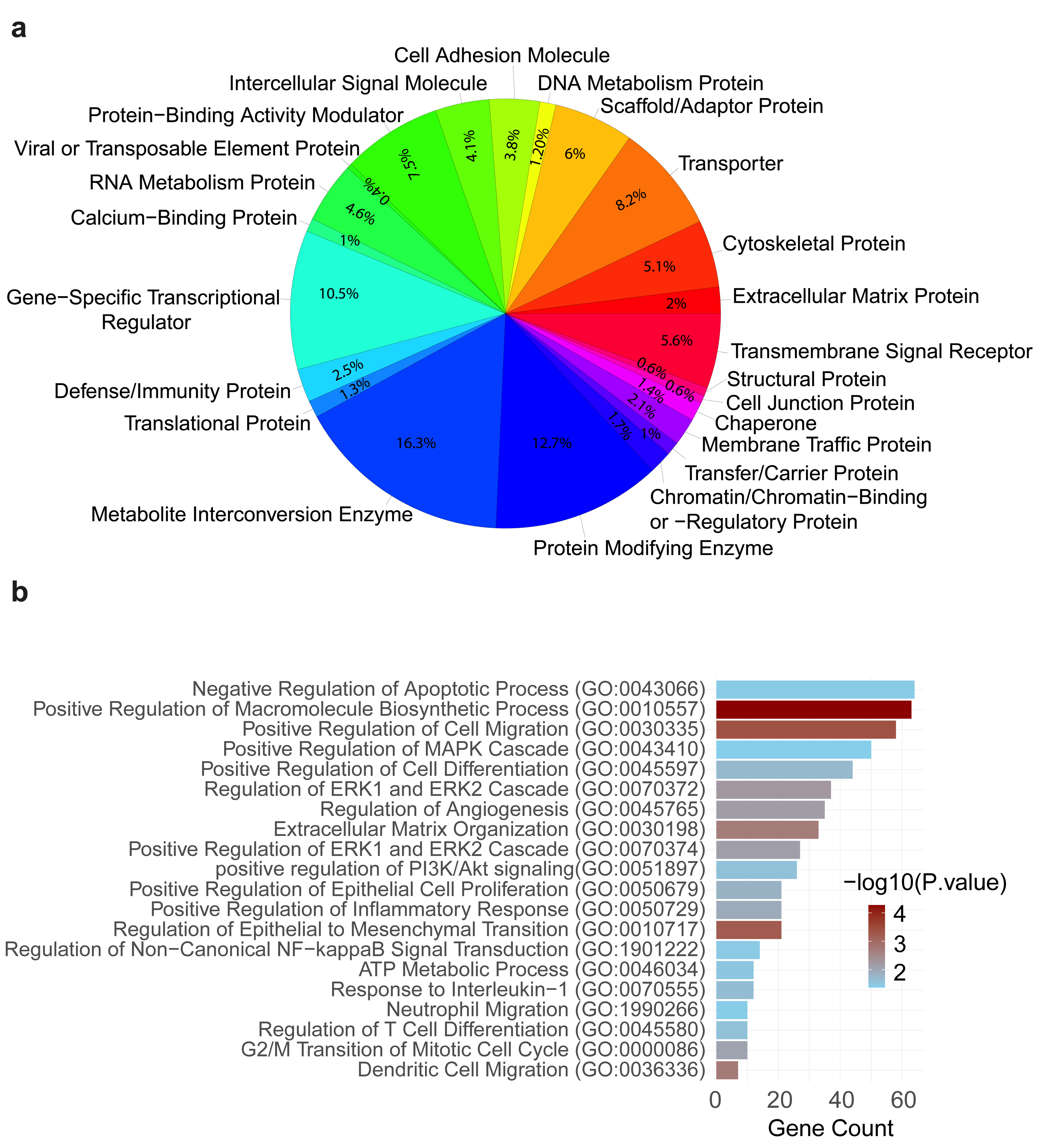
**

**Figure S3**. **Whole transcriptomic analysis of MC38 and MC38-SH3 tumors. (a)** Pie chart showing differentially expressed genes (DEGs) classified functionally based on gene ontology. The global set of DEGs between MC38 and MC38-SH3 tumors was classified into functional gene categories to highlight their biological roles using the PANTHER Classification System (<http://pantherdb.org/>). **(b)** Over-representation analyis of DEGs to identify major gene ontology biological (GO-BP) processes. Bar length represents the total gene count and color intensity (from dark to light) reflects the log10(p-value).

**
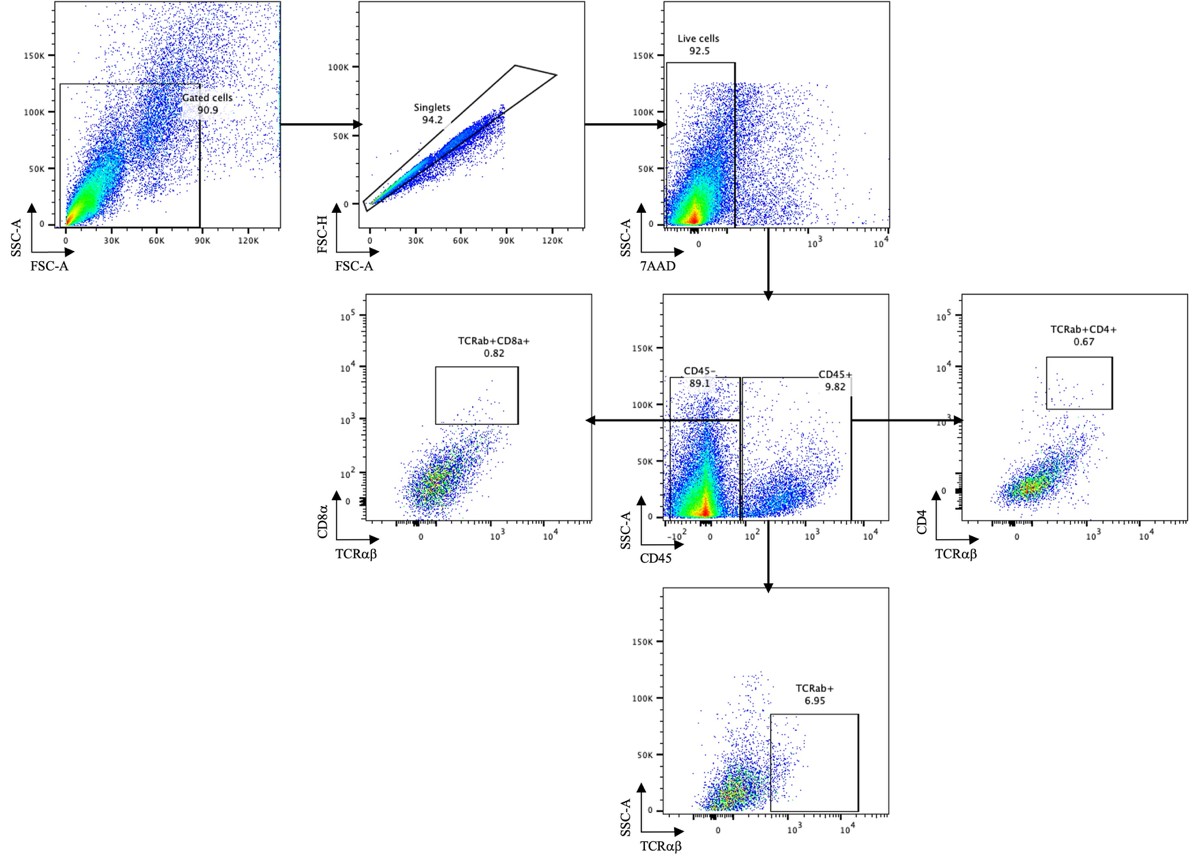
**

**Figure S4. The gating strategy used for identification of the intratumoral T cell populations.** Single-cell suspensions of tumors were stained with different fluorophore-conjugated antibodies and analyzed by flow cytometry. Following the exclusion of doublets and non-viable cells, immune cells were identified as positive for the CD45 marker. Among the CD45^+^ cells, the T cell populations were identified by staining with mAbs to TCRαβ and CD4 or CD8 cell surface markers.


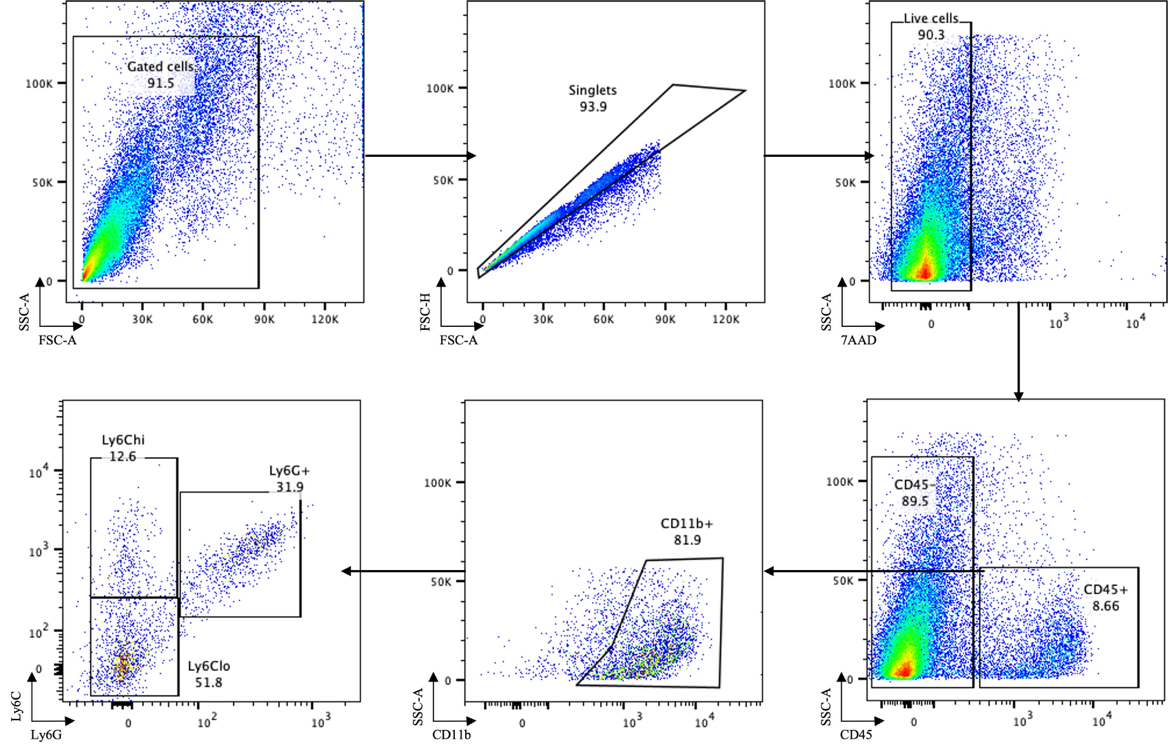


**Figure S5. The gating strategy used for identification of the intratumoral myeloid cell populations.** Single-cell suspensions were stained with different fluorophore-conjugated antibodies and analyzed by flow cytometry. Following the exclusion of doublets and non-viable cells, immune cells were identified as positive for the CD45 marker. Among the CD45^+^ cells, the myeloid cells (CD11b^+^) were gated to identify granulocytic cells (Ly6G^+^), Ly6C^hi^ monocytes and Ly6C^lo^ macrophages.

## **Supplementary Materials and Methods**

## Generation of MCJ-deficient cell lines

The siRNA construct targeting MCJ was generated using the human H1 RNA polymerase III promoter cloned into the pCAG-GFP vector upstream of the cytomegalovirus enhanced green fluorescent protein (EGFP) cassette to obtain the pSuper-EGFP vector. The siRNA for MCJ, 5′-gatccccGCGAGAGGCTAGTCTTATTttcaagagaAATAAGACTAGCCTCTCGCtttttggaaa-3′, was cloned into the BglII and HindIII sites downstream of the H1 promoter. The murine MCJ target sequences are shown in capital letters. The final construct, pCAG-EGFP-Super-mouse-siMCJ, was transfected into MC38 cells using Lipofectamine 2000 (ThermoFisher Scientific, Waltham, MA), as recommended by the manufacturer. For stable transfection, the above-described transfection procedure was followed and clones were selected in medium containing 400 μg/ml of G418 (Life Technologies, Inc., Carlsbad, CA), as described previously (1). Transfection efficiency was validated by fluorescence microscopy, detecting green fluorescence in transfected cells, and by Western blots to quantify the level of MCJ protein.

Western blot analysis

Whole-cell lysates were prepared utilizing RIPA lysis buffer, as previously described (2). Equal volumes of lysates were loaded onto a 4-20% gradient Bis-Tris Precast gel (Cat# M00656, GenScript Biotech, Piscataway, NJ) and subjected to electrophoretic transfer onto PVDF membranes. The membranes were incubated overnight at 4°C with a MCJ-specific monoclonal antibody (a gift from Dr. Mercedes Rincon) or with antibodies specific to various components of the ETC (Cat# 42642, Cell Signaling Technology, Danvers, MA): NDUFAB1 and NDUFS1 (Complex I), CYC1 (Complex III), COX1/MT-CO1 (Complex IV), and β-actin (Cat# 8H10D10, Cell Signaling). The secondary antibodies employed were goat anti-mouse-HRP and goat anti-rabbit HRP (Cell Signaling), as appropriate. The resulting blots were developed using Pierce ECL Plus (ThermoFisher Scientific) and imaged using the Azure chemiluminescence imaging system in accordance with the manufacturer’s protocol. The blot images were analyzed using Image J software (Version 1.54p) and the recorded band intensity was utilized to calculate the relative fold change between control and experimental samples. All proteins of interest were normalized over β-actin before calculating the relative fold change.

Cell proliferation assay

The impact of MCJ knockdown on cellular proliferation was assessed by manual cell counting of in vitro-cultured cells over a period of 72 hours. Cells were seeded in 24-well plates at a density of 25,000 cells per well in duplicates and incubated for different time points (0, 24, 48, and 72 hours). At each time point, cells were detached by trypsinization, resuspended in fresh culture medium, and mixed with an equal volume of 0.4% Trypan Blue solution. Cells were counted manually under a light microscope at 10X magnification.

Mitochondrial and Glycolytic Stress Tests

Oxygen consumption rate (OCR) and extracellular acidification rate (ECAR) were measured using the Seahorse XF Mito Stress Test and Glycolytic Stress Test kits (Agilent), respectively, using 10-20x10^3^ cells/well. For these studies, OCR and ECAR were determined for MC38 cells in comparison with MC38-SH3 and MC38-Con cell lines. For the Mito Stress Test, OCR was assessed under basal conditions and following sequential injections of oligomycin (1.5 µM), FCCP (0.75 µM), and rotenone/antimycin A (0.5 µM each) in Seahorse XF assay medium consisting of DMEM supplemented with 10 mM glucose, 2 mM glutamine, and 1 mM pyruvate (pH 7.4), according to the manufacturer’s instructions. For the Glycolytic Stress Test, ECAR was assessed under basal conditions and following sequential injections of glucose (10 mM), oligomycin (1.5 µM), and 2-deoxy-D-glucose (2-DG; 50 mM) in Seahorse XF assay medium consisting of DMEM supplemented with 2 mM glutamine and 1 mM pyruvate (pH 7.4), according to the manufacturer’s instructions. All assays were performed using the Agilent Seahorse XFe96 Extracellular Flux Analyzer.

Experimental animals

C57BL/6 mice were purchased from the Jackson Laboratory (Bar Harbor, ME).  All animals were bred in the animal facility of the College of Medicine and Health Sciences, United Arab Emirates University. Athymic NMRI/nude^nu/nu^ mice were purchased from Charles River Laboratories (Sulzfeld, Germany) and housed in filtered-air laminar flow cabinets, as described (3). Male mice were used at 8–12 weeks of age. Studies involving animals were performed in accordance with and after approval of the Animal Research Ethics Committee of the United Arab Emirates University (Protocols #ERA_2018_5743 and ERA_2024_4434).

*In vivo* tumor experiments

C57BL/6 mice were randomly divided into two experimental groups, and then subcutaneously injected in the right flank with either 1x10^5^ MC38 or MC38-SH3 tumor cells. For studies involving nude mice, the number of tumor cells implanted was 2x10^6^ cells per mouse. Tumor growth was monitored regularly by palpation and measured twice weekly using digital calipers. Tumor volume was calculated using the formula: Volume=W^2^/(2xL), where the width represents the perpendicular dimension and the length represents the longest dimension. Mice were sacrificed on day 30 after inoculation. Tumors were isolated and used for further analysis.

Flow cytometry

Tumors were finely minced and enzymatically digested in gentleMACS C-tubes (Miltenyi Biotec, Germany), using a tumor dissociation kit (Miltenyi Biotec, Bergisch Gladbach, Germany) and the gentleMACS dissociator, according to the manufacturer’s instructions. Analysis of tumor single cells was carried out using multi-color flow cytometry. Washed cells were incubated with FcγR blocking antibody (anti-mouse CD16/32) (Cat# 101302, Biolegend, San Diego, CA) for 30 mins at 4°C; non-viable cells were excluded using 7-AAD viability dye (Biolegend). Cells were stained with fluorochrome-conjugated primary antibodies at pre-determined optimum concentrations for 30 min at 4°C in the dark. The following antibodies (purchased from Biolegend, San Diego, CA, USA) were used in the current study: anti-CD45-PE (Cat# 103106), anti-CD45-APC (Cat# 103112), anti-TCRαβ-BV785 (Cat# 109249), anti-CD8α-APC/Fire 750 (Cat# 100766), anti-CD4-FITC (Cat# 100509), anti-CD11b-Alex Fluor-488 (Cat# 101217), anti-CD11c-PE (Cat# 117308), anti-Ly6C-APC/Fire 750 (Cat# 128045), anti-Ly6G-APC (Cat# 127614), and anti-MHC I H-2K^b^-BV421 (Cat# 116525). For some experiments, anti-MHC I H-2D^b^-FITC (Cat# 11-5999-82; eBioscience) was used. Data were acquired on a BD FACSCelesta flow cytometer (BD Biosciences, Mountain View, CA, USA) and analyzed using FlowJo v10 (BD Biosciences).

Immunohistochemistry (IHC) staining

Formalin-fixed paraffin-embedded tumor blocks were sectioned into 4-5 μm thick sections using a rotary microtome (Shandon AS325, USA). Sections were mounted on Aminopropylsilane (APS)-coated slides and dried overnight at 37°C. IHC staining, deparaffinization, rehydration, and endogenous peroxidase activity were performed per established protocols in our laboratory (4, 5). Sections were incubated overnight at 4°C with the following primary antibodies: anti-CD8 (Cat# ab209775; Abcam, Cambridge, UK) and anti-granzyme B (Cat# 44153S; Cell Signaling). Following washing, tissue sections were treated with goat polyclonal secondary antibody (HRP polymer) (Cat# ab214882; Abcam) for 45 minutes at room temperature. After washing, DAB (3, 3'-diaminobenzidine) chromogen (Dako, Carpinteria, CA) was applied to detect HRP activity, with hematoxylin used for counterstaining. Imaging was conducted using an Olympus BX51 microscope model V-LH100HG (Olympus Corporation, Japan) at 40x magnification. Positive cells were counted across 15-20 randomly selected high-power fields (HPF), and the average count was determined.

Quantitative real-time PCR

qRT-PCR was performed as described previously (3, 6). Pre-made TaqMan primers and probes were utilized to study the expression of the following target genes in whole tumor tissue extracts; IFN-γ (Mm01168134_m1), Perforin-1(Mm00812512_m1), Granzyme B (Mm00442834_m1), S100A9 (Mm00656925_m1), HIF1α (Mm00468869_m1), and Arginase 1 (Mm00475988_m1); these primers were obtained from Applied Biosystems (Foster City, CA). The mRNA levels of target genes were normalized to the housekeeping gene hypoxanthine guanine phospho-ribosyltransferase (HPRT; Mm01545399_m1) using the comparative ΔCq method. The expression of the target gene is reported as the level of expression relative to HPRT and presented as fold change relative to expression in tumors from mice inoculated with parental MC38 cells.

Whole Transcriptome Sequencing

RNA was extracted from in vitro cultured MC38 and MC38-SH3 cells, as well as from single-cell suspensions of tumor tissue harvested from in vivo experiments, using TRIzol reagent (Invitrogen), followed by purification with the RNeasy Mini Kit (Qiagen, Valencia, CA), as previously described (6). RNA quality and quantity were assessed using a NanoDrop ND-1000 spectrophotometer (Thermo Scientific, Waltham, MA). For RNA sequencing, RNA integrity was assured by determining the RIN (RNA Integrity number) of each sample using an Agilent 4200 TapeStation (Santa Clara, CA). RNA with RIN ≥8 was used for RNA sequencing. Messenger RNA was purified from total RNA using poly-T oligo-attached magnetic beads. After fragmentation, the first strand cDNA was synthesized using random hexamer primers followed by the second strand cDNA synthesis. The library was ready after end repair, A-tailing, adapter ligation, size selection, amplification, and purification. The cDNA library was checked with Qubit and real-time PCR for quantification and bioanalyzer for size distribution detection. Sequencing was carried out on NovaSeq 6000 PE150 using the NovaSeq 6000 V1.5 sequencing kit.

Bioinformatics Analysis

RNAseq Data Preprocessing. For RNAseq analysis, the raw reads often contain low-quality reads or those with adapters, potentially compromising subsequent analyses. To address this, we employed a rigorous raw reads filtering process: (i) Eliminating reads with adapters; (ii) Removing reads with over 10% undetermined bases (N); and (iii) Excluding low-quality reads with a Quality value (Qscore) of ≤ 5 for over 50% of the bases. For mapping, we utilized HISAT2 in our analysis workflow. We directly mapped reads to the transcriptome when focusing on obtaining the differential expression of significant genes. Conversely, for studies involving alternative splicing, variations, or fusion gene detection, we opted for genome-level alignment. This approach enables precise positioning of a significant number of effective junction reads, crucial for capturing comprehensive RNAseq data. The abundance of transcripts directly mirrors gene expression levels in RNAseq experiments. Gene expression is estimated by the count of sequencing-mapped transcripts to the genome or exon. The read count is proportionate to gene expression, gene length, and sequencing depth, providing a holistic measure of gene expression levels.

Differential Expression Analysis and Feature Selection. The read counts were optimized for alignment against the mouse reference genome mm39 and used as input for differential expression analysis of MC38 (as control) and MC38-SH3 cells (as MCJ-deficient transfectant) using R with the DESeq2 package (7). The base mean read count, fold change, p-value, and q-value (Benjamini-Hochberg adjusted) are derived from this analysis. Next, we employed the lfcShrink() function to facilitate the shrinkage of log fold changes. By utilizing this function, we were able to enhance the accuracy and reliability of our log fold change estimates.

In the initial analysis of differentially expressed genes (DEGs) from in vitro grown tumor cells, we selected genes with a significance threshold of adjusted p-value ≤ 0.05 and an absolute log2 fold change > 1.5, identifying genes with substantial expression changes between the two conditions (control vs. MCJ-deficient). In contrast, for whole tumor transcriptomic analysis, where cellular heterogeneity may dilute gene expression differences, we used a more inclusive threshold (adjusted p-value ≤ 0.05 and |log2FC| > 0.0) to capture subtle but biologically relevant changes associated with the tumor microenvironment. These results were visualized in a volcano plot generated using the ggplot2 package in R. To further investigate expression patterns, we generated a heatmap of the top 100 DEGs (50 upregulated and 50 downregulated) using the ComplexHeatmap package in R (8). Raw counts were first normalized using DESeq2’s median-of-ratios method, followed by row-wise z-score transformation to standardize expression levels across genes.

Functional and Pathway Enrichment Analysis of DEGs. To identify biologically relevant pathways and functions, we performed over-representation analysis (ORA) on DEGs from MC38 and MC38-SH3 cancer cell lines as well as whole tumor tissues. ORA was conducted using Gene Ontology Biological Processes (GO:BP) and hallmark gene sets (MH: hallmark) from the MSigDB database (9). The term "hallmark geneset" refers to a curated collection derived by integrating multiple founder sets to robustly represent distinct biological pathways. ORA is a statistical approach that detects whether predefined gene sets are significantly enriched in a subset of genes (e.g., DEGs) compared to random chance. ORA was performed using the **Enrichr** tool (10) (as per 17 April 2025). Statistically significant enrichments were defined by a p-value < 0.05 and a minimum of 5 genes per gene set (minGeneset >5) to ensure robust biological interpretation. The results were visualized as a bar graph generated with the ggplot2 package in R, where the x-axis represents the total gene count, and the color of the bars indicates statistical significance, represented as -log10(p-value).

Furthermore, we used transcriptomic profiling of whole tumor tissues to perform pathway enrichment analysis on the identified DEGs using Gene Set Variation Analysis (GSVA), enabling the assessment of pathway-level activity changes across samples (11). GSVA is a specialized gene set enrichment method designed for single-sample analysis, allowing pathway-centric exploration of molecular data. It shifts the focus from individual genes to gene sets, providing a more powerful and intuitive approach to functional analysis. In the GSVA package, we specifically used the z-score method, along with its unique GSVA algorithm, to normalize gene expression values across samples, enhancing the sensitivity in detecting sample-level pathway activity changes. This combination enables a robust, non-parametric analysis of pathway enrichment that accounts for biological variability across individual tumor samples.

Next, we calculated GSVA enrichment scores using a subset of Hallmark gene sets (MH) from the Molecular Signatures Database (MSigDB v2023.2.Mm) (9), a collection that represents well-defined biological pathways and sets the minimum and maximum gene set size to 02 and 500, respectively. Given the known function of MCJ, a deficiency in this protein would be expected to alter mitochondrial function and metabolic pathways, thereby influencing tumor behavior. Accordingly, we focused on genes and pathways related to mitochondrial function, using a curated collection of mitochondrial genes (MGs) obtained from MitoCarta(v. 3.0) (12), UniProt (13), and Human Protein Atlas (orthologous genes) (14). These mitochondrial genes were then compared with the total identified DEGs, resulting in 174 genes (88 upregulated and 86 downregulated) common to both sets, which were selected for pathway overrepresentation analysis.

Immune Cell Infiltration. To gain a deeper understanding of the TME, we utilized ImmuCellAI-mouse, a robust computational tool designed to predict the relative abundance of 36 immune cell subtypes in various mouse tissue samples based on gene expression data (15). The tool estimates immune cell infiltration by calculating the ssGSEA enrichment score of expression deviation profiles for each cell type. The tool utilizes a reference profile generated from 1502 immune cell samples, with the median gene expression value per cell type, ensuring high prediction accuracy. Moreover, the tool facilitates the analysis of immune cell infiltration differences across diverse sample groups. When comparing two groups, the tool performs a Mann-Whitney U-test, while for multiple groups, an ANOVA test is conducted, offering a comprehensive statistical framework for evaluating immune cell distribution across different disease conditions.

Correlation analysis of MCJ (*DNAJC15*) expression in human cancers

We utilized TCGAplot (16), an R package designed for the visualization and analysis of TCGA datasets, to investigate the expression patterns of *DNAJC15* across 33 cancer types. Expression levels in tumor and corresponding normal tissues were compared, and statistical significance was evaluated using the Wilcoxon test with a threshold of P < 0.05. Furthermore, we examined the promoter methylation status of DNAJC15. DNA methylation levels were quantified using β values, which range from 0 (unmethylated) to 1 (fully methylated). Based on established thresholds, promoter regions with higher β values were considered hypermethylated, whereas lower β values were classified as hypomethylated. Furthermore, we used the UALCAN webserver (17) to analyze the protein expression level of DNAJC15 based on data from the Clinical Proteomic Tumor Analysis Consortium (CPTAC) and the International Cancer Proteogenome Consortium (ICPC) datasets. Next, a diagnostic receiver operating characteristic (ROC) curve was used to evaluate the ability of *DNAJC15* expression to discriminate colon adenocarcinoma (COAD) tumor samples from normal colon tissues. The ROC curve plots the true positive rate (sensitivity) against the false positive rate (1-specificity) across a range of DNAJC15 expression cut-off values. The area under the ROC curve (AUC) quantifies the overall diagnostic performance: an AUC of 0.5 indicates no discriminatory ability, whereas an AUC closer to 1.0 reflects excellent diagnostic accuracy.

Differential Gene Expression and TMB/MSI Correlation in High and Low *DNAJC15* Groups

TCGA-COAD tumor samples were stratified into high-MCJ and low-MCJ groups based on the median expression of DNAJC15. The top 100 DEGs from each group were visualized using a heatmap. Furthermore, correlations between high- and low-MCJ expression–associated gene sets and tumor mutation burden (TMB) as well as microsatellite instability (MSI) were analyzed across multiple cancer types. Pearson correlation coefficients were calculated and visualized using a radar chart. This analysis highlights distinct associations between MCJ expression status and TMB/MSI across cancers, providing insights into the potential role of MCJ in tumor immunogenicity and genomic instability.

Correlation analysis of the High & low MCJ groups with immune-related genes

We performed a comprehensive correlation analysis between MCJ and various immune-related genes in human COAD tumors, including immune checkpoint genes (ICGs), chemokines (*CCL1-CCL28, CX3CL1, CXCL1-CXCL17*), chemokine receptors (*CCR1-CCR10, CXCR1-CXCR6, XCR1, CX3CR1*), immune stimulators (*CD27, CD40LG, ICOS, TNFRSF/TNFSF* family members), and immune inhibitors (*PDCD1/PDCD1LG2, CTLA4, LAG3, TIGIT, IDO1*). Additionally, we also examined associations with immune cell infiltration patterns and immune scores

Recurrence-Free Survival Analysis of *DNAJC15* in COAD Patients

Recurrence-free survival (RFS) analysis of DNAJC15 expression was performed using the Kaplan–Meier (KM) Plotter tool (18). Patients were stratified based on DNAJC15 expression using the lower quartile (25th percentile) as the cutoff. All available follow-up data were included, with censoring applied at the defined threshold. Survival analyses were conducted for the entire COAD patient cohort as well as for the subset of patients who did not receive chemotherapy. Differences in RFS between groups were evaluated using the log-rank test, with a p-value < 0.05 considered statistically significant.

**References**

1. Hatle KM, Neveu W, Dienz O, Rymarchyk S, Barrantes R, Hale S, Farley N, Lounsbury KM, Bond JP, Taatjes D, Rincon M. 2007. Methylation-controlled J protein promotes c-Jun degradation to prevent ABCB1 transporter expression. *Mol Cell Biol* 27: 2952-66

2. Aryappalli P, Al-Qubaisi SS, Attoub S, George JA, Arafat K, Ramadi KB, Mohamed YA, Al-Dhaheri MM, Al-Sbiei A, Fernandez-Cabezudo MJ, Al-Ramadi BK. 2017. The IL-6/STAT3 Signaling Pathway Is an Early Target of Manuka Honey-Induced Suppression of Human Breast Cancer Cells. *Front Oncol* 7: 167

3. Kaimala S, Mohamed YA, Nader N, Issac J, Elkord E, Chouaib S, Fernandez-Cabezudo MJ, Al-Ramadi BK. 2014. Salmonella-mediated tumor regression involves targeting of tumor myeloid suppressor cells causing a shift to M1-like phenotype and reduction in suppressive capacity. *Cancer Immunol Immunother* 63: 587-99

4. Fernandez-Cabezudo MJ, Faour I, Jones K, Champagne DP, Jaloudi MA, Mohamed YA, Bashir G, Almarzooqi S, Albawardi A, Hashim MJ, Roberts TS, El-Salhat H, El-Taji H, Kassis A, O'Sullivan DE, Christensen BC, DeGregori J, Al-Ramadi BK, Rincon M. 2016. Deficiency of mitochondrial modulator MCJ promotes chemoresistance in breast cancer. *JCI Insight* 1: e86873

5. Masad RJ, Idriss I, Mohamed YA, Al-Sbiei A, Bashir G, Al-Marzooq F, Altahrawi A, Fernandez-Cabezudo MJ, Al-Ramadi BK. 2024. Oral administration of Manuka honey induces IFNgamma-dependent resistance to tumor growth that correlates with beneficial modulation of gut microbiota composition. *Front Immunol* 15: 1354297

6. Al-Saafeen BH, Al-Sbiei A, Bashir G, Mohamed YA, Masad RJ, Fernandez-Cabezudo MJ, Al-Ramadi BK. 2022. Attenuated Salmonella potentiate PD-L1 blockade immunotherapy in a preclinical model of colorectal cancer. *Front Immunol* 13: 1017780

7. Love MI, Huber W, Anders S. 2014. Moderated estimation of fold change and dispersion for RNA-seq data with DESeq2. *Genome Biol* 15: 550

8. Gu Z. 2022. Complex heatmap visualization. *Imeta* 1: e43

9. Liberzon A, Birger C, Thorvaldsdottir H, Ghandi M, Mesirov JP, Tamayo P. 2015. The Molecular Signatures Database (MSigDB) hallmark gene set collection. *Cell Syst* 1: 417-25

10. Kuleshov MV, Jones MR, Rouillard AD, Fernandez NF, Duan Q, Wang Z, Koplev S, Jenkins SL, Jagodnik KM, Lachmann A, McDermott MG, Monteiro CD, Gundersen GW, Ma'ayan A. 2016. Enrichr: a comprehensive gene set enrichment analysis web server 2016 update. *Nucleic Acids Res* 44: W90-7

11. Hanzelmann S, Castelo R, Guinney J. 2013. GSVA: gene set variation analysis for microarray and RNA-seq data. *BMC Bioinformatics* 14: 7

12. Rath S, Sharma R, Gupta R, Ast T, Chan C, Durham TJ, Goodman RP, Grabarek Z, Haas ME, Hung WHW, Joshi PR, Jourdain AA, Kim SH, Kotrys AV, Lam SS, McCoy JG, Meisel JD, Miranda M, Panda A, Patgiri A, Rogers R, Sadre S, Shah H, Skinner OS, To TL, Walker MA, Wang H, Ward PS, Wengrod J, Yuan CC, Calvo SE, Mootha VK. 2021. MitoCarta3.0: an updated mitochondrial proteome now with sub-organelle localization and pathway annotations. *Nucleic Acids Res* 49: D1541-D7

13. UniProt C. 2021. UniProt: the universal protein knowledgebase in 2021. *Nucleic Acids Res* 49: D480-D9

14. Thul PJ, Lindskog C. 2018. The human protein atlas: A spatial map of the human proteome. *Protein Sci* 27: 233-44

15. Miao YR, Xia M, Luo M, Luo T, Yang M, Guo AY. 2022. ImmuCellAI-mouse: a tool for comprehensive prediction of mouse immune cell abundance and immune microenvironment depiction. *Bioinformatics* 38: 785-91

16. Liao C, Wang X. 2023. TCGAplot: an R package for integrative pan-cancer analysis and visualization of TCGA multi-omics data. *BMC Bioinformatics* 24: 483

17. Chandrashekar DS, Karthikeyan SK, Korla PK, Patel H, Shovon AR, Athar M, Netto GJ, Qin ZS, Kumar S, Manne U, Creighton CJ, Varambally S. 2022. UALCAN: An update to the integrated cancer data analysis platform. *Neoplasia* 25: 18-27

18. Gyorffy B. 2024. Integrated analysis of public datasets for the discovery and validation of survival-associated genes in solid tumors. *Innovation (Camb)* 5: 100625
